# *VvEPFL9-1* Knock-Out via CRISPR/Cas9 Reduces Stomatal Density in Grapevine

**DOI:** 10.3389/fpls.2022.878001

**Published:** 2022-05-17

**Authors:** Molly Clemens, Michele Faralli, Jorge Lagreze, Luana Bontempo, Stefano Piazza, Claudio Varotto, Mickael Malnoy, Walter Oechel, Annapaola Rizzoli, Lorenza Dalla Costa

**Affiliations:** ^1^Research and Innovation Centre, Fondazione Edmund Mach, San Michele all’Adige, Italy; ^2^Global Change Research Group, San Diego State University, San Diego, CA, United States; ^3^Department of Viticulture and Enology, University of California Davis, Davis, CA, United States; ^4^Department of Geography, University of Exeter, Exeter, United Kingdom

**Keywords:** *Vitis vinifera*, stomata, genome editing, climate change, water-use efficiency

## Abstract

Epidermal Patterning Factor Like 9 (EPFL9), also known as STOMAGEN, is a cysteine-rich peptide that induces stomata formation in vascular plants, acting antagonistically to other epidermal patterning factors (EPF1, EPF2). In grapevine there are two *EPFL9* genes, *EPFL9-1* and *EPFL9-2* sharing 82% identity at protein level in the mature functional C-terminal domain. In this study, CRISPR/Cas9 system was applied to functionally characterize *VvEPFL9-1* in ‘Sugraone’, a highly transformable genotype. A set of plants, regenerated after gene transfer in embryogenic calli *via Agrobacterium tumefaciens*, were selected for evaluation. For many lines, the editing profile in the target site displayed a range of mutations mainly causing frameshift in the coding sequence or affecting the second cysteine residue. The analysis of stomata density revealed that in edited plants the number of stomata was significantly reduced compared to control, demonstrating for the first time the role of EPFL9 in a perennial fruit crop. Three edited lines were then assessed for growth, photosynthesis, stomatal conductance, and water use efficiency in experiments carried out at different environmental conditions. Intrinsic water-use efficiency was improved in edited lines compared to control, indicating possible advantages in reducing stomatal density under future environmental drier scenarios. Our results show the potential of manipulating stomatal density for optimizing grapevine adaptation under changing climate conditions.

## Introduction

Drought is a threat to the quality and yield of grapevine in the world’s important wine grape growing regions (Mosedale et al., 2016; Van Leeuwen and Destrac-Irvine, 2017; Van Leeuwen et al., 2019). These regions are expected to have decreased precipitation with associated risks of developing soil water deficit in coming years (IPCC, 2014; Sherwood and Fu, 2014; Scholasch and Rienth, 2019). One adaptation strategy seen in plants to tolerate water limitation involves stomatal regulation of water loss (Hunt et al., 2010; Hughes et al., 2017; Bertolino et al., 2019; Caine et al., 2019; Dayer et al., 2020; Gambetta et al., 2020). Stomata are pores mainly located in the leaf epidermis. The opening of these pores controls leaf gas exchange (CO_2_ uptake for photosynthesis and water loss *via* transpiration) and is regulated by changes in turgor pressure in the guard cells surrounding these pores. The two guard cells respond to a range of environmental signals, often in conflict with each other, and sometimes rapidly changing (e.g., humidity, CO_2_ concentration, light). In drought-stressed grapevine, stomatal closure is triggered by hydraulic signals and maintained by abscisic acid following re-watering (Lovisolo et al., 2010; Tombesi et al., 2015). Genotypic variation for stomatal sensitivity to reduced water availability has been shown to exist in grapevine (Schultz, 2003; Soar et al., 2006; Bota et al., 2016; Villalobos-González et al., 2019; Faralli et al., 2021).

Stomatal density and distribution in the epidermal tissue also plays a critical role in determining transpiration rate per unit of leaf area (Hunt et al., 2010). Previous work focusing on natural variation for stomatal anatomical features provided evidence of a close negative relationship between plant water-use efficiency and stomatal density (Bertolino et al., 2019; Faralli et al., 2019). According to extensive studies carried out in Arabidopsis (Doheny-Adams et al., 2012; Franks et al., 2015; Hepworth et al., 2015; Lee et al., 2015), stomatal density and distribution are under the control of small cysteine-rich peptides (CRP) called epidermal patterning factors (EPFs) highly conserved in a wide range of higher plants (Lu et al., 2019). Three members of this family play a key role in the formation of stomata: EPF1, EPF2 and EPFL9. EPF2 and EPF1 are expressed in the epidermis, in the earlier and later stages of leaf development, respectively. EPF2 inhibits the formation of cells considered the precursors of stomata guard cells, while EPF1 inhibits the subsequent differentiation of these same precursors and induces asymmetric cell division (Hara et al., 2009). Epidermal Patterning Factor Like 9 (EPFL9), also known as STOMAGEN, plays an antagonist role with respect to EPF1 and EPF2 as it induces stomata formation (Kondo et al., 2010). EPF-peptides interact with two transmembrane receptors of epidermal cells, ERECTA and Too Many Mouths (TMM). While EPF1 and EPF2 activate the receptor complex which in turn induces a MAPKs (Mitogen-Activated Protein Kinases) cascade (Morales-Navarro et al., 2018; Zoulias et al., 2018) leading to the destabilization of important transcription factors involved in the formation of stomata (SPEECHLESS, MUTE, FAMA; Pillitteri et al., 2007; Chen et al., 2020), STOMAGEN inactivates it. STOMAGEN is the only known positive regulator of stomata produced in mesophyll, and was confirmed to act independently of EPF1 and EPF2 (Hunt et al., 2010; Kondo et al., 2010; Sugano et al., 2010; Ohki et al., 2011). Its activity is antagonized by that of EPF2, however, it is not well understood if the antagonistic action is due to the sharing of an identical binding site in the common receptor or to other mechanisms (Ohki et al., 2011). An evolutionary model suggests that EPFL9 may derive from the duplication of EPF1/2 with a subsequent alteration in the function (Shimada et al., 2011). This is confirmed by the fact that EPF1/2 are more widespread in higher plants compared to EPFL9 (Lu et al., 2019). Despite the different amino acid composition among the CRP different sub-classes and across species, the members of CRPs have in common a small size, a conserved N-terminal region that include an apoplast secretion signal and a functional C-terminal domain containing cysteine residues (Marshall et al., 2011).

Several functional genomics studies, based on the ectopic expression or silencing of EPF1, EPF2, or EPFL9, have recently demonstrated a highly conserved functional paradigm in Arabidopsis and cereals. In barley, Hughes et al. (2017) proved that *HvEPF1* overexpression limits stomatal development. In a hexaploid bread wheat, Dunn et al. (2019) decreased stomatal density (SD) *via* the overexpression of *TaEPF1* and *TaEPF2* orthologues and demonstrated improvements in water-use efficiency without affecting yield when SD reduction was moderate. Similarly, in rice Caine et al. (2019) and Mohammed et al. (2019) elucidated the function of *OsEPF1* adopting an over-expression approach. Adding to the studies on rice, Lu et al. (2019) confirmed the role of *OsEPF1*, *OsEPF2* and *OsEPF9* by a dual strategy, both over-expression and down-regulation *via* RNA interference. Yin et al. (2017) were the first to apply the genome editing technology in rice to disrupt *OsEPFL9*.

Gene editing *via* the clustered regularly interspaced short palindromic repeats (CRISPR)/CRISPR-associated protein 9 (Cas9) (Jinek et al., 2012) is to date the most powerful tool for functional genomics studies in plants (Liu et al., 2016). CRISPR/Cas9 system can efficiently produce nucleotide mutations into precise positions in the genome through the combined action of a specific guide RNA and the Cas9 nuclease which cleaves the DNA eliciting the non-homologous end-joining (NHEJ) pathway for DNA repair (Podevin et al., 2013). NHEJ may produce knock-out (KO) mutants with random insertion or deletion (indels) of variable lengths at the Cas9 cleavage site causing frameshift mutations or loss of amino acids in protein-coding sequences. These KO mutants are perfect systems to prove the function of a candidate gene (Jain, 2015). This technology is steadily boosting (Hess et al., 2017; Anzalone et al., 2019) and, coupled with the advancements of *in-vitro* culture practices, represents a knowledge-based strategy for the genetic improvements of cultivated plants, with relevant advantages compared to traditional breeding (Chen et al., 2019).

In grapevine, CRISPR/Cas9 technology has been successfully applied to evaluate the function of genes involved in susceptibility or tolerance to diseases, mainly caused by fungal pathogens (Malnoy et al., 2016; Giacomelli et al., 2019; Li et al., 2020; Wan et al., 2020; Chen et al., 2021; Scintilla et al., 2021), or to enhance tolerance to cold stress (Wang et al., 2021).

In this study, we inactivated *VvEPFL9-1* in a grapevine table grape variety, ‘Sugraone’, adopting a genome editing approach based on CRISPR/Cas9 technology. Different edited lines with a significant reduction in stomatal density were produced and three of them were analyzed to investigate how reducing stomatal density affects grapevine physiological performance under different environmental conditions.

## Materials and Methods

### Search for the Orthologous Gene of *AtEPFL9* in Grapevine Genomic Databases and Experimental Confirmation in a Set of Grapevine Genotypes

*AtEPFL9* sequence (AT4G12970) was used as a query to interrogate the publicly available genomic databases of *Vitis* spp. (Supplementary Table 1). To experimentally confirm the presence of two *VvEPFL9* paralogs in a set of grapevine genotypes, DNA was extracted from leaf tissue of ‘Chardonnay’, ‘Merlot’, ‘Syrah’, ‘Cabernet Sauvignon’, ‘Touriga National’, ‘Pinot Noir clone Entav 115’, ‘Pinot Noir PN40024’, ‘Sugraone’ and ‘Riparia Glorie de Montpellier’ using Nucleospin Plant II kit (Macherey–Nagel, Düren, Germany) following the manufacturer’s instruction. Genomic DNA was quantified using Nanodrop 8,800 (Thermo Fischer Scientific, Waltham, MA, United States) and diluted to a final concentration of 30 ng/μL. Two PCR reactions were performed in 25 μl final volume containing 1 × PCR BIO (Resnova, Rome, Italy), 30 ng of genomic DNA and 0.5 μM of primers in order to amplify *VvEPFL9-1* (primer VvEPFL9-1_fw and VvEPFL9-1_rv, see Supplementary Table 2) and *VvEPFL9-2* (primer VvEPFL9-2_fw and VvEPFL9-2_rv, see Supplementary Table 2). Amplification products were checked on agarose gel, purified using CleanNGS magnetic beads (CleanNA, Waddinxveen, Netherlands) and sequenced by Sanger sequencing (FEM Sequencing Platform Facility, San Michele all’Adige, Italy). Sequencing outputs were analyzed with Blast online tool^1^ and for the alignment of the sequences the software MEGAX (Kumar et al., 2018) was used.

### Plant Material (Gene Transfer Experiments, *in-vitro* and Greenhouse Growth)

The CRISPR/Cas9 binary vector with the customized sgRNA was purchased from DNA Cloning Service (Hamburg, Germany). The nucleotide sequence of *SpCAS9* and of *NPTII* genes were codon optimized for the plant expression system and their sequences are available on the company website.^2^ The sequence of the guide RNA carried by the vector was designed with CRISPR-P 2.0 software^3^ and recognizes a region of 20 bp in the third exon of *VvEPFL9-1* (GCACATACAATGAATGCAAA, on-score = 0.7058). *Agrobacterium tumefaciens* (A.t.)-mediated gene transfer was performed on embryogenic calli of ‘Sugraone’ according to Dalla Costa et al. (2022). *NPTII* was used as selectable marker to confer resistance to kanamycin. Regenerated plants were screened by PCR for the presence of *SpCAS9* (to select plants which integrated T-DNA) in 20 μl final volume containing 1 × PCR BIO (Resnova, Rome, Italy), 0.5 μM of each primer (SpCAS9_Fw and SpCAS9_Rv, see Supplementary Table 2) and 30 ng of genomic DNA. DNA was extracted from freshly frozen leaf tissue (approximately 100 mg) using Nucleospin Plant II kit (Macherey–Nagel, Düren, Germany) following the manufacturer’s instruction, quantified using Nanodrop 8,800 (Termo Fischer Scientific, Waltham, MA, United States) and diluted to a final concentration of 30 ng/μL.

Edited lines and WT control were propagated *in-vitro* in sterilized jars containing WP medium (McCown and Lloyd, 1981) in a growth chamber at 100 photosynthetic photon flux density (PPFD) ± 20 (μmol m^−2^ s^−1^), 24°C and a 16/8 light/dark photoperiod. Four biological replicates of healthy developed edited lines and of the WT control were acclimatized in the greenhouse using 0.25 l plastic pots with three holes in the bottom to allow for water drainage, filled with a similar amount of growing substrate (Extra quality - Semina, TerComposti, Calvisano, Italy) and covered by parafilm on the top. Plants were kept in a growth chamber (PPFD 100 +/− 20 μmol m^−2^ s^−1^, 24°C, 16/8 light/dark photoperiod) and after 1 week, holes were gradually made in the top of the parafilm over the course of 2 weeks. After 17 days, plants were repotted into 0.75 l pots all containing growing substrate (Extra quality - Special Cactus, TerComposti, Calvisano, Italy). Pots were kept in the same growth chamber for a subsequent 10 days before moving to the greenhouse. In the greenhouse, plants were grown under natural light supplemented by high-pressure sodium lamps system (PPFD 200–250 μmol m^−2^ s^−1^) with a 16-h/8-h light–dark photoperiod. Environmental conditions including temperature and humidity during the growth chamber and greenhouse cultivation are shown in Supplementary Figure 1.

### Molecular Characterization of Edited Lines

#### Transgene Copy Number Quantification

The quantification of *SpCAS9* copy number (CN) in grapevine lines was carried out according to real-time PCR method developed by Dalla Costa et al. (2009). Reactions were performed in a 96-well plate on a C1000 thermal cycler (Bio-Rad, Hercules, United States) equipped with CFX96 real-time PCR detection system (Bio-Rad, Hercules, United States). The real-time PCR singleplex reaction was carried out in a 10 μl final volume containing 1 × SsoAdvanced Universal Probes Supermix (Bio-Rad, Hercules, United States), 40 ng of genomic DNA, 0.3 μM primers (Sigma, Haver hill, UK) and a 0.2 μM specifc Taqman probe (Sigma, Haverhill, UK). The thermal protocol was as follows: polymerase activation for 3 min at 95°C followed by 40 cycles of denaturation of 10 s at 95°C, annealing of 5 s at 58°C and 5 s at 60°C and an elongation of 30 s at 72°C. Primers and Taqman probes used to amplify grapevine endogenous *VvCHI* (VvChiRT_fw; VvChiRT_rv; VvChiRT_Probe) and *SpCAS9* (SpCas9RT_fw; SpCas9RT_rv; SpCas9RT_Probe) were reported in Supplementary Table 2. The standard curves (four points, starting from 10^6^ plasmid molecules and adopting a serial dilution of 1:5) were built with a plasmid pGEM-T easy (Promega, Madison, Wisconsin, United States), in which we cloned a fragment of *VvCHI* and *SpCAS9.* For each sample, the *SpCAS9* CN was calculated using the following formula: (transgene total copies / endogenous gene total copies) × 2. The total copies of transgene and endogenous gene were calculated on the basis of the mean values of the quantification cycles (Cq) of two technical replicates.

#### On- and Off-Target Editing Evaluation

In the grapevine lines integrating T-DNA, a region of the gene *VvEPFL9-1* containing the site targeted by the sgRNA/Cas9 complex, was amplified with primers VvEPFL9-1_fw and VvEPFL9-1_rv (see Supplementary Table 2) both elongated with overhang Illumina adapters. PCR was carried out in 20 μl final volume containing 1 × PCR BIO (Resnova, Rome, Italy), 0.4 μM of each primer and 30 ng of genomic DNA. The Illumina library was sequenced on an Illumina MiSeq (PE300) platform at the Sequencing Platform Facility of Fondazione Edmund Mach (San Michele all’Adige, Italy). CRISPResso2 pipeline^4^ (Clement et al., 2019) was used to process the raw paired end reads with default parameters and to visualize the mutations profiles in the target sequences. For the analysis of the off-target site in the gene *VvEPFL9-2*, a PCR was carried out in 25 μl final volume containing 1 × PCR BIO (Resnova, Rome, Italy), 0.5 μM of each primer (VvEPFL9-2_fw and VvEPFL9-2_rv, see Supplementary Table 2) and 30 ng of genomic DNA. Amplification products were checked on agarose gel, purified using CleanNGS magnetic beads (CleanNA, Waddinxveen, Netherlands) and sequenced by Sanger sequencing (FEM Sequencing Platform Facility). Sequencing outputs were analyzed with Blast online tool.^5^

#### T-DNA Integration Site Identification

T-DNA integration points (IP) were determined following the method described in Dalla Costa et al. (2020). The library was sequenced by Illumina MiSeq (PE300) platform at the Sequencing Platform Facility of Fondazione Edmund Mach (San Michele all’Adige, Italy). The putative genomic regions identified were validated by PCR amplification. PCR was performed in a 20 μl final volume containing 1 × PCR BIO (Resnova, Rome, Italy), 40 ng of genomic DNA and 0.5 μM of the primers reported in Supplementary Table 2. Amplification products were checked on agarose gel, purified using PureLink Quick Gel Extraction (Invitrogen, Carlsbad, CA, United States) and sequenced by Sanger sequencing (FEM Sequencing Platform Facility). Sequencing outputs were analyzed with the Blast sequence server (using the database PN40024.v4_REF_genome) available online at the European network INTEGRAPE website.^6^

### Experimental Conditions and Physiological Analysis

#### Experiment 1: Well-Watered (WW) Conditions in Greenhouse

Biological replicates of edited lines S-*epfl9*KO1 (*n* = 4) and S-*epfl9*KO2 (*n* = 4), and of ‘Sugraone’ WT (*n* = 4) kept in a greenhouse for 2 months were used. Pots were covered in aluminum foil and wrapped in plastic to limit soil evaporation (Supplementary Figure 2). All plants were measured daily for 14 days at the same time each morning for mass of water loss.

#### Experiment 2: Water-Stress (WS) Conditions in Greenhouse

The same plants used in Experiment 1 were used in Experiment 2. Control pots (soil-filled pots without plants) were placed at the end of each row in randomized positions, weighed by balance and returned to the same positions every day to assess soil evaporation. Pots dried down naturally for a subsequent 15 days.

#### Experiment 3: Well-Watered (WW) Conditions in an Automated High-Throughput Phenotyping Platform

Biological replicates of the edited line S-*epfl9*KO6 (*n* = 6) and ‘Sugraone’ WT (*n* = 4), maintained in greenhouse for 12 months, with a height range of 60–70 cm and a weight brought to 3,000 g (in 5 l pots) were used. Plants were moved inside the phenotyping platform (WIWAM, Ghent, Belgium) at the Plant Phenotyping Facility of Fondazione Edmund Mach where temperature was set to 28/25°C, photoperiod to 16/8 h and average PPFD to 300 μmol m^−2^ s^−1^ at apical leaf level. Plants were automatically watered every day at 6:00 AM to target weight (3,000 g) and pot weight was evaluated before and after watering for 12 days.

#### Soil Water Content, Transpiration, and Leaf Area Determination

In Experiment 1 and 2, total transpirable soil water (TTSW) was calculated as the difference between pot mass at day 1, fully watered (100% capacity), and the pot mass at the end of the natural dry down when transpiration reached a minimum. Fully watered plants (100% relative soil water content) were weighted after watering to capacity and allowing pots to drain for 2 h. The fraction of transpiration soil water (FTSW) was calculated as a daily ratio between the amount of soil water remaining in the pot left for transpiration and the TTSW using the equation: FTSW = (PMn – PMfinal)/TTSW, where PMn is the pot mass for each day, and PMfinal is the pot mass at the end of the day 11. FTSW data were reported in Supplementary Figure 3. At day 12 (i.e., after Experiment 1), plants were unwrapped from the aluminum and plastic coverings, re-watered to 100% of their initial weight using syringes and weighed as a starting mass for the stress application. In both Experiment 1 and 2, transpiration (g/cm^2^) was measured as the grams of water lost daily, normalized by the relative leaf area for each individual [T = (mass 0 - mass 1)/relative leaf area, where 0 and 1 represent the days in consecutive order]. Growth was measured as a relative leaf area every other day for a period of 28 days using RGB imaging. The software Easy Leaf Area (Easlon and Bloom, 2014) was used for analysis. Photos of the plants were taken at the same distance and tripod angle (45°) to provide uniform and consistent assessment of relative leaf area (example in Supplementary Figure 4A). A biomass-leaf area estimated curve was constructed using eight plants of varying sizes validating the non-destructive approach (Supplementary Figure 5). In Experiment 3, daily water-use was automatically calculated as daily pot weight loss (g). In addition, projected leaf area (pixels) was calculated at the beginning and at the end of the experiment (day 1 and day 12 respectively) as the average green pixels in four RGB images collected at different pot angles and analyzed with the WIWAM software (example in Supplementary Figure 4B).

#### Stomatal Characterization

Samples for stomatal characterization were taken under well-watered conditions as well as at the end of the drought treatment (i.e., Experiment 1 and 2). Leaves were chosen with the same size and position, typically leaf three, unless abnormal. Clear gel nail polish was applied to the abaxial and adaxial surfaces of the leaf to create an imprint of the leaf surface and allowed to dry. Clear tape was used to peel off the nail polish, and the tape was mounted on a microscope slide. Slides were imaged using a compound microscope (DM, Leica Microsystems, Wetzlar, Germany) at 40x and at five different technical positions of the same area (0.3 mm^2^) on the four biological replicates for a total of twenty measurements of stomata density per individual. Stomatal size (SS) was characterized from three technical replicates from three biological replicates for a total of 9 replicates per individual. These 9 replicates were averaged to create an average radius (r) for reach individual, and the stomatal size was subsequently calculated as $SS=0.5\pi r^{2}\;$ ; stomatal size is equal to 0.5 multiplied by the average length of stomata squared multiplied by π.

#### Gas-Exchange Analysis, SPAD and Leaf Temperature

For Experiment 1, 2 and 3, gas-exchange measurements were carried out using a portable infra-red gas analyzer and a 2 cm^2^ leaf cuvette with an integral blue–red LED light source (LiCOR 6,400-40XT, Lincoln, NE, United States). Inside the cuvette, flow rate was set at 400 μmol s^− 1^, leaf temperature at 24°C, PPFD to 1,500 μmol m^−2^ s^−1^ and *C_a_* of 400 μmol mol^−1^. In Experiment 1, measurements of the response of photosynthesis (*A*) to sub-stomatal CO_2_ concentrations (*C_i_*) curves (*A*/*C_i_*) were performed between 9:00 and 12:00, on the most expanded leaf from each plant. For *A/C_i_*, *C_a_* was sequentially decreased to 300, 200, 150, 75 and 50 μmol mol^−1^ before returning to the initial concentration of 400 μmol mol^−1^. This was followed by a sequential increase to 500, 700, 900, 1,100, 1,300, and 1,500 μmol mol^−1^. Readings were recorded when *A* reached steady state. The maximum velocity of Rubisco for carboxylation (*V_cmax_*) and the maximum rate of electron transport demand for Ribulose 1,5-bisphosphate (RuBP) regeneration (*J_max_*) were estimated as described by (Duursma, 2015; Easlon and Bloom, 2014). *A_sat_* represents CO_2_ assimilation rate at saturating PPFD while *g_s_* represents stomatal conductance at ambient CO_2_ (*C_a_*). Intrinsic water-use efficiency (*_i_WUE*) was calculated as = *A_sat_* / *g_s_*. During Experiment 2, measurements of *A* and *g_s_* were taken every day on fully expanded leaves for the first 3 days to record a baseline gas-exchange before water stress was applied. Subsequently gas-exchange data were recorded every 2 days in fully expanded leaves. In Experiment 3, gas-exchange parameters (*A* and *g_s_*), leaf temperature and leaf chlorophyll content were measured at day 5 on the same leaves, respectively with LiCOR 6,400-40XT (Lincoln, NE, United States), an infra-red thermometer (62 MAX+, FLUKE Corporation, Everett, Washington, United States) and a SPAD (Minolta SPAD 502).

#### Carbon Isotope Composition

Carbon isotope composition was estimated in leaves with the same leaf size and position, count as leaf three unless abnormal. Samples for stomatal characterization were taken first, and the remaining fresh leaf tissue was dried at 80°C for 2 days to be used for δ^13^C determination. δ^13^C was analyzed in 2 mg aliquots of leaf sample weighed in tin capsules. Samples were combusted in an elemental analyzer (Thermo Flash EA 1112 Series, Bremen, Germany), CO_2_ was separated by chromatography and directly injected into a continuous-flow isotope ratio mass spectrometer (Thermo Finnigan Delta V, Bremen, Germany) through the interface ConFlo IV dilutor device (Thermo Finningan, Bremen, Germany). Samples were measured in duplicate. The isotope ratios were expressed in δ‰ against Vienna-Pee Dee Belemnite for δ^13^C according to the following equation: δ ‰ = ( $R_{SA}-R_{REF})/R_{REF}$ where *R_SA_* is the isotope ratio measured for the sample and *R_REF_* is the international standard isotope ratio. The isotopic values were calculated using a linear equation against working in-house standards, which were themselves calibrated against the international reference materials L-glutamic acid USGS 40 (US Geological Survey, Reston, VA, United States), fuel oil NBS-22 and IAEA-CH-6. The uncertainty of measurement (calculated as 2 standard deviations) was 0.1‰.

### Statistics

Statistical analyses were performed using R software (R Core Team, 2020). A one-way ANOVA was used to compare differences in cumulative transpiration, conductance, photosynthesis, and water use efficiency between edited and WT lines for each day of measurement. *Post hoc* comparisons using Fisher’s LSD test were carried out to assess group differences. *p* values lower than 0.05 were considered significant.

## Results

### Identification of *AtEPFL9* Orthologous Genes in Grapevine

**Figure 1:**
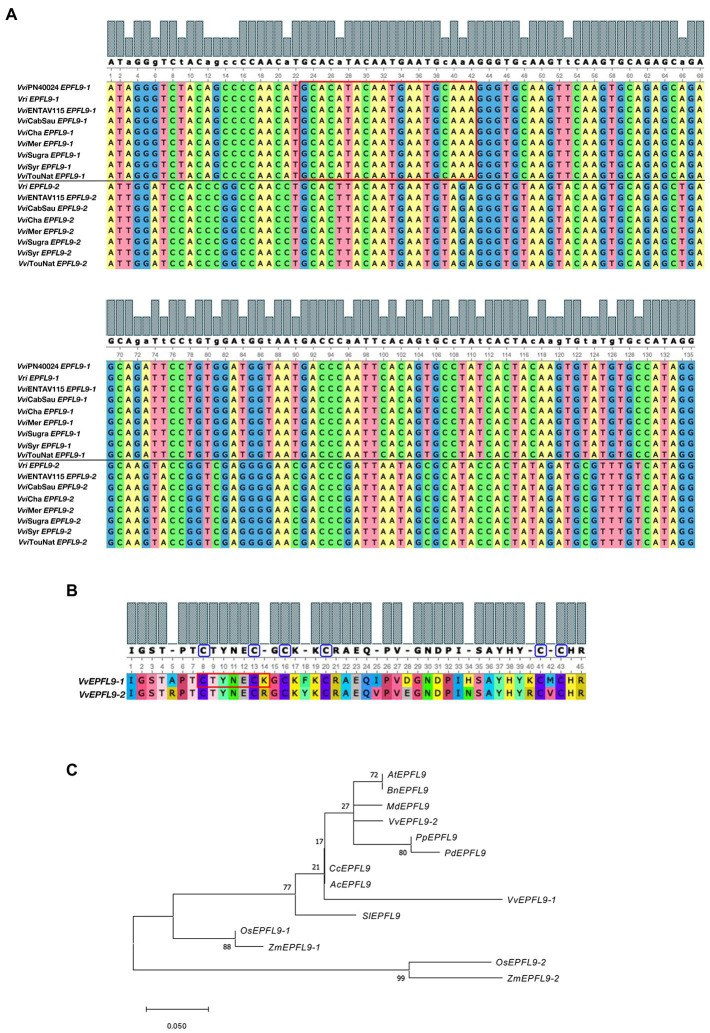
Analysis of *VvEPFL9* paralogs. **(A)** Alignment of the nucleotide sequence encoding for the C-terminal domain (135 bp) obtained by Sanger sequencing of PCR fragments amplified on genomic DNA with primers VvEPFL9-1_fw; VvEPFL9-1_rv and VvEPFL9-2_fw; VvEPFL9-2_rv (see primer list in Supplementary Table 2). Genomic DNA was extracted from leaves of ‘Pinot Noir PN40024’, *Vitis riparia* ‘Riparia Glorie de Montpellier’, ‘Pinot Noir clone Entav 115’, ‘Cabernet Sauvignon’, ‘Chardonnay’, ‘Merlot’, ‘Sugraone’, ‘Syrah’, ‘Touriga National’. The red rectangle indicates the 20 bp-target site recognized by the sgRNA/Cas9 complex. **(B)** Alignment of the C-terminal protein domain of VvEPFL9-1 and VvEPFL9-2, translated from the 135 bp nucleotide sequences shown in **(A)**. Cysteine residues are circled in blue. The red rectangle indicates the peptide region corresponding to the target site. **(C)** Phylogenetic tree of the Arabidopsis *AtEPF9* mature peptide and its orthologs from some dicotyledonous (*Brassica napus*, *Malus × domestica*, *Vitis vinifera*, *Prunus persica*, *Prunus domestica*, *Prunus dulcis, Citrus clementina, Actinidia chinensis, Solanum lycopersicum*) and monocotyledonous (*Orytia sativa*, *Zea mays*) plant species. The alignments were generated with MUSCLE (MEGA X) and visualized with Unipro UGENE [http://ugene.net/faq.html (Okonechnikov et al., 2012)]. The phylogenetic tree was built with MEGA X using Maximum Likelihood (1,000 replicates bootstrap). Accession Numbers: *VvEPFL9-1* (*Vitis vinifera*; Vitvi05g01370); *VvEPFL9-2* (*Vitis vinifera*; contig VV78X057312.8. BioProject PRJEA18357); *AtEPFL9* (*Arabidopsis thaliana*; AT4G12970); *BnEPFL9* (*Brassica napus*; BnaA08g04900D-1); *OsEPFL9-1* (*Oryza sativa*; BGIOSGA005039-TA); *OsEPFL9-2* (*Oryza sativa*; BGIOSGA026626-TA); *ZmEPFL9-2* (*Zea mays*; Zm00001d049795_T001); *ZmEPFL9-1* (*Zea mays*; Zm00001d012079_T001); *SlEPFL9* (*Solanum lycopersicum*; Solyc08g066610.3.1); *MdEPFL9* (*Malus domestica*; mRNA:MD10G0128800); *CcEPFL9* (*Citrus clementina*; ESR50459); *PpEPFL9* (*Prunus persica*; ONH92727); *PdEPFL9* (*Prunus dulcis*; VVA33635); *AcEPFL9* (*Actinidia chinensis*; PSR86312).

Two *VvEPFL9* gene variants (hereinafter *VvEPFL9-1* and *VvEPFL9-2*) were found in contigs of publicly available genomes of different *Vitis vinifera* varieties and of some other species within the same genus (*Vitis sylvestris*, *Vitis arizonica*, *Vitis riparia*; Supplementary Table 1). In the last annotation of the PN40024 grapevine reference genome (PN40024.v4.1,^7^ genome assembly version 12X.v4) *VvEPFL9-1* (Vitvi05g01370) was localized on chromosome 5 (position 20,461,188–20,461,813) while VvEPFL9-2 (Vitvi07g04390) on chromosome 7 (position 17,537,397–17,536,742). Interestingly, before the new version of reference genome and related annotation was made publicly available (INTEGRAPE Workshop, 2021) in November 2021, only *VvEPFL9-1* was localized on the genome while the position of *VvEPFL9-2* was not assigned (VCost.v3 annotation). According to gene prediction, *VvEPFL9-1*/*−2* coding sequence have a length of about 330/315 bp and are composed of three exons encoding for: an N-terminal region with a secretion signal for the apoplast [i.e., first 27 amino acid according to SignalP-5.0 software (Almagro Armenteros et al., 2019)^8^ a central region likely involved in the processing of the mature peptide and a C-terminal domain of 45 amino acids containing 6 conserved cysteines, that is the functional peptide. A check on genomic DNA extracted from a panel of genotypes (i.e., ‘Pinot Noir PN40024’, ‘Riparia Glorie de Montpellier’, ‘Pinot Noir clone Entav 115’, ‘Cabernet Sauvignon’, ‘Chardonnay’, ‘Merlot’, ‘Sugraone’, ‘Syrah’ and ‘Touriga National’), confirmed the presence of both gene variants in all the analysed samples with a very high conservation among genotypes (Supplementary Table 3). In all the genotypes no SNPs were detected between the two alleles of both isoforms in the region coding for the functional domain, except in Cabernet Sauvignon where an allelic polymorphism in position 25 was detected in *VvEPFL9-1*, which leads to two different amino acids after the first cysteine of the array (serine or threonine, both polar uncharged). Considering only the region encoding for the C-terminal domain (135 bp), the identity between the two variants was 74%, with a large part of polymorphism leading to synonymous codons (Figure 1A). At the protein level, the alignment of the C-terminal domains encoded by the two variants showed an identity of 82%, with 8 out of 45 different amino acids (Figure 1B). In five positions (14, 25, 28, 40, and 42) substitutions are conservative, i.e., the pair of amino acids belong to the same class, while in the remaining three positions (5, 18, and 34) the substitutions are non-conservative. A comparison with *AtEPFL9* mature peptide revealed that the identity between *VvEPF9-1* and *AtEPFL9* is 82% while the identity between *VvEPF9-2* and *AtEPFL9* is 95% (Supplementary Figure 6). Moreover, the relationship of *VvEPFL9-1/−2* with the orthologues of some di- and monocotyledonous plant species including some perennial fruit trees (retrieved from Ensembl Plants genomic database),^9^ is shown in Figure 1C.

### The Knock-Out of *VvEPF9-1* Reduces Stomatal Density in Grapevine

**Figure 2:**
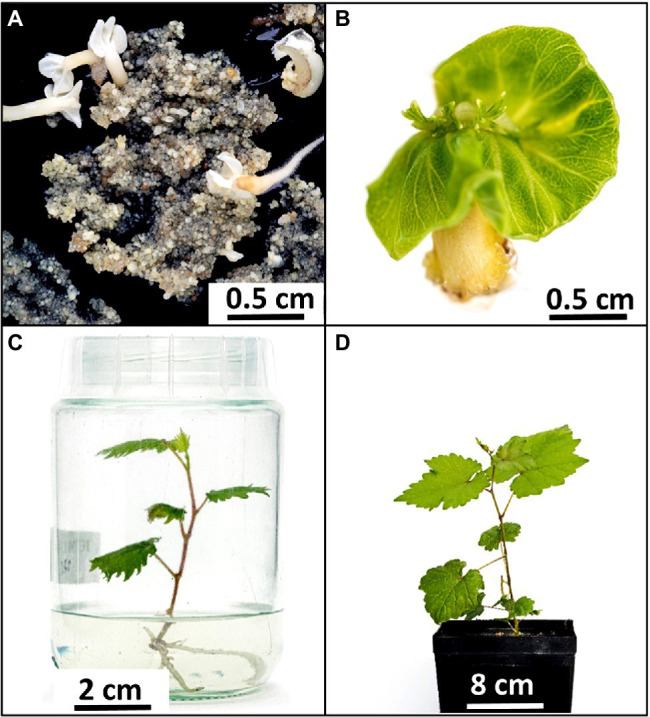
Pipeline to obtain *epfl9*-1 mutants for physiological characterization. **(A)** Embryogenic callus of ‘Sugraone’ 7 months after co-cultivation with *Agrobacterium tumefaciens*. Some embryos are developing on a homogeneous callus mainly formed by small globular embryos. **(B)** Embryo producing shoot. **(C)** *In-vitro* plantlet cultivated in baby jar. **(D)** Greenhouse plant after 2 months from acclimatization of an *in-vitro* plantlet.

**Figure 3:**
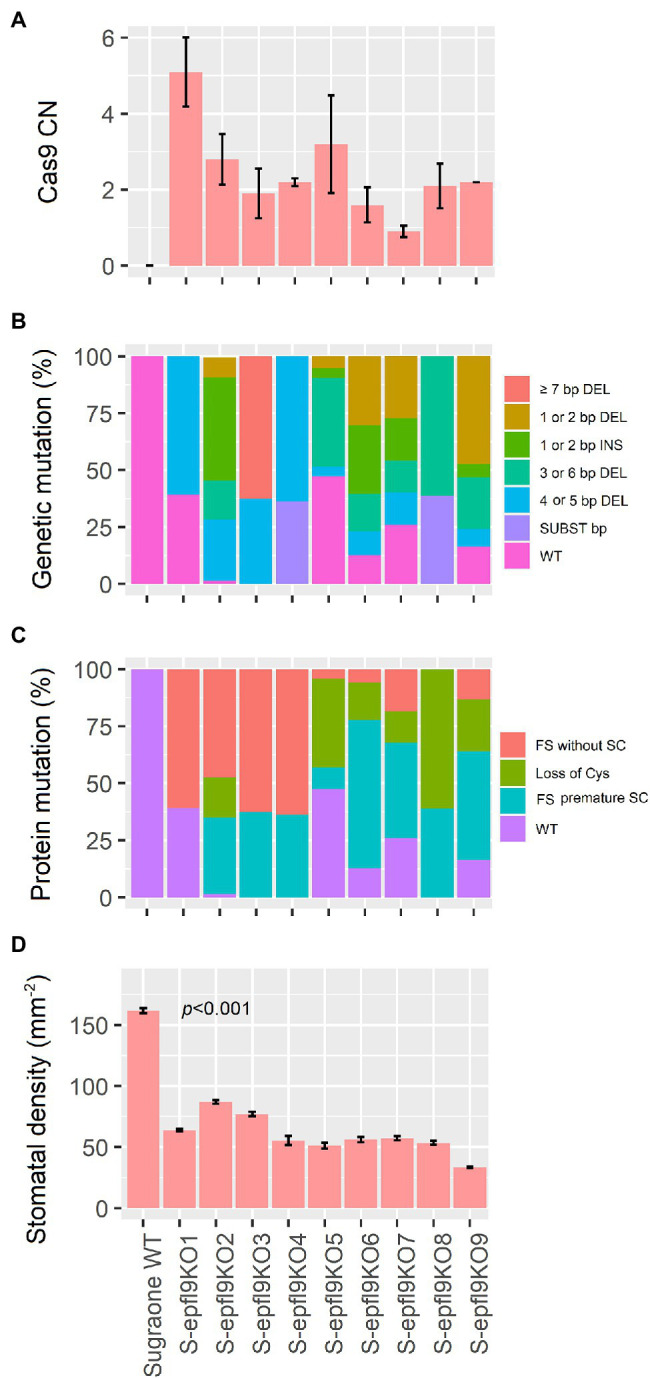
Characterization of 9 ‘Sugraone’ transgenic lines. **(A)** Quantification of *SpCas9* copy numbers (CN) integrated in the plant genome. CN were calculated by Real-time PCR as the mean value of two measurements obtained for two *in-vitro* biological replicates (except for line S-*epfl9*KO9 for which only one value is available). **(B)** Bar plot indicating the mutation profile in the genomic target site on exon 3 of *VvEPF9-1* after CRISPR/Cas9 editing. The mutation pattern and rate (%) of a specific mutation (IN/DEL, insertion/deletion and SUB, substitution) were determined by the number of reads calculated by Illumina sequencing (see Supplementary Table 4). Different kinds of mutations are indicated with a different color. WT = the wild-type sequence. **(C)** Bar plot indicating the resulting mutation profile in the functional mature VvEPF9-1 peptide, predicted according to the nucleotide mutations in B (see Supplementary Table 4). The different outcomes at protein level are indicated with a different FIGURE 3color. FS, frameshift mutations; SC, stop codons. **(D)** Measurements of stomatal density in the third leaf from the apex. For each plant, four leaves from four biological replicates were analyzed, each in five different technical positions of the same area for a total of twenty measurements.

A highly transformable genotype of *Vitis vinifera*, ‘Sugraone’ was used for gene transfer of the CRISPR/Cas9 machinery in order to obtain edited plants knocked-out for the *VvEPF9-1* gene. The sgRNA was designed to target a region of 20 nucleotides in the third exon, spanning across “TGC” triplets coding for the first and the second cysteine of the functional C-terminal domain (Figures 1A,B; Supplementary Table 4). In particular, the cleavage operated by Cas9 was expected to affect the “TGC” triplet coding for the second cysteine, this being located 3 nucleotides upstream of the PAM site (i.e., GGG; Figure 1A). The corresponding region of *VvEPF9-*2 has 3 mismatches compared with the target site on *VvEPF9-1*, in positions 6, 18 and 20, the last two in the seed region close to the PAM site (Figure 1A). Several shoots were regenerated from somatic embryos after 7–10 months from *Agrobacterium tumefaciens* co-culture (Figure 2), and nine of them were selected for molecular characterization. The Cas9 integration copy number varied in the transgenic lines, ranging from 1 integration copy for line S-*epfl9*KO7 to 5 integration copies for line S-*epfl9*KO1, with the majority of lines showing values close to one or two copies (Figure 3A). A T-DNA integration site was identified for 5 lines: S-*epfl9*KO1 (chr18: position 2,096,753), S-*epfl9*KO2 (chr01: position 4,310,437), S-*epfl9*KO3 (Chr13: position 5,599,304), S-*epfl9*KO6 (chr04: position 6,948,780), S-*epfl9*KO7 (chr03: position 405,924). Concerning T-DNA rearrangements, all the lines showed a trimming of several bases at the LB border, ranging from 31 bp of S-*epfl9*KO7 to 110 bp of line S-*epfl9*KO3, and a T-DNA tandem repeat was detected in line S-*epfl9*KO1. The analysis of the genomic “on-target” site in *VvEPF9-1* proved that all lines were edited, some completely while others showed a degree of wild-type target sequence, indicated as WT (Figure 3B; Supplementary Table 4). In general, the editing profile was highly heterogeneous, with a composite mutation profile for many lines (e.g., S-*epfl9*KO2, S-e*pfl9*KO5, S-*epfl9*KO6, S-*epfl9*KO7, S-*epfl9*KO9), including deletions of increasing size (from 1 bp to more than 7 bp), insertions of 1 or 2 bp, and single base substitutions. The most frequent kind of mutations were deletions of 4 or 5 bp (Figure 3B). The resulting mutations in the protein sequence were frameshift mutations (FS) with or without the formation of premature stop codons (SC), or non-frameshift mutations with loss of the second cysteine due to deletion of 3 or 6 bp or to a single base substitution (Figure 3C). The analysis of stomatal density in leaves of greenhouse-cultivated plants (2 months old) showed a significant reduction in stomata number in transgenic lines compared to WT (Figure 3D). This reduction was significant even for the lines maintaining a remarkable rate of non-mutated *VvEPF9-1* (i.e., S-*epfl9*KO1, S-*epfl9*KO5, S-e*pfl9*KO7) and for lines that went through the loss of the second cysteine of the 6-Cys-array, highlighting the crucial role of such residue (i.e., S-*epfl9*KO5 and S-*epfl9*KO8). The editing in the potential “off-target” site in *VvEPFL9-2* was assessed and no mutations were found in all the transgenic lines (Supplementary Figure 7). This proved that 3 mismatches with respect to the sgRNA, 2 of which close to the PAM site, were enough to avoid Cas9 unspecific cleavage at this site. *In vitro* and greenhouse edited plants did not show phenotypic defects due to pleiotropic effects (e.g., rate of growth, total leaf area, chlorophyll content) compared to the control plants (data not shown).

**Figure 4:**
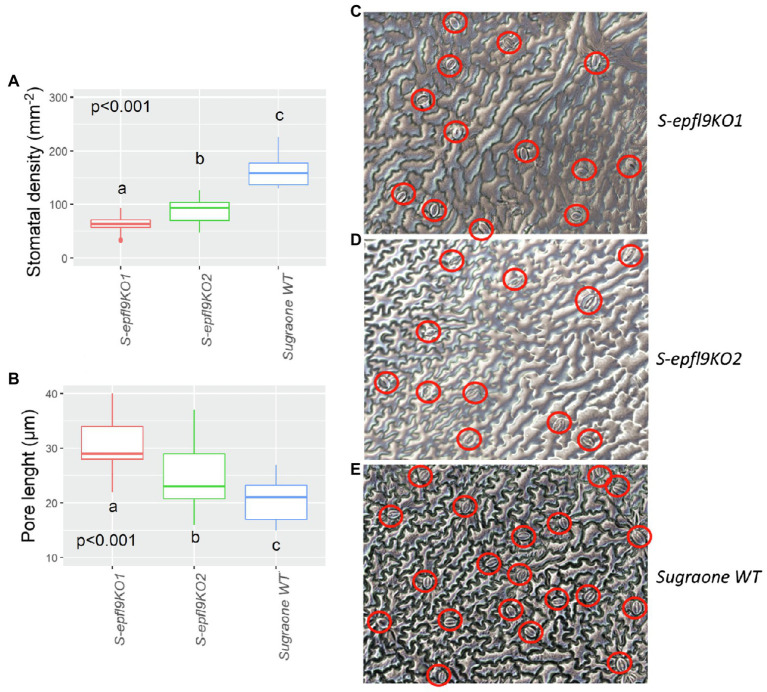
Characterization of stomata in selected *epfl9-1* knock-out mutants. **(A)** Stomatal density for S-*epfl*9KO1, S-*epfl9*KO2 and Sugraone WT. **(B)** Pore length for S-*epfl9*KO1, S-*epfl9*KO2 and Sugraone WT. Whiskers indicate the ranges of the minimum and maximum values. Data were analysed with one-way ANOVA (*n* = 6–9). Different letters indicate significantly different values according to Fisher’s test. **(C–E)** Images of nail polish printing of leaf tissue, respectively, from S-*epfl9*KO1, S-*epfl9*KO2 and Sugraone WT.

Analysis of stomatal anatomical features confirmed the significant differences for stomatal density and pore length between the selected S-*epfl9*KO1 and S-*epfl9*KO2 knock-out mutants and WT (Figure 4). S-*epfl9*KO1 had an average SD of 65 stomata mm^−2^ while SD for S-*epfl9*KO2 was 95 stomata mm^−2^, both significantly lower values than that of ‘Sugraone’ WT (160 stomata mm^−2^) respectively by 60 and 40%. Conversely, pore length was significantly higher in S-*epfl9*KO1 and S-*epfl9*KO2 than ‘Sugraone’ WT, by up to 30%.

### The Knock-Out of *VvEPF9-1* Enhances Plant Water Use Efficiency Under Optimal Growth Conditions

**Figure 5:**
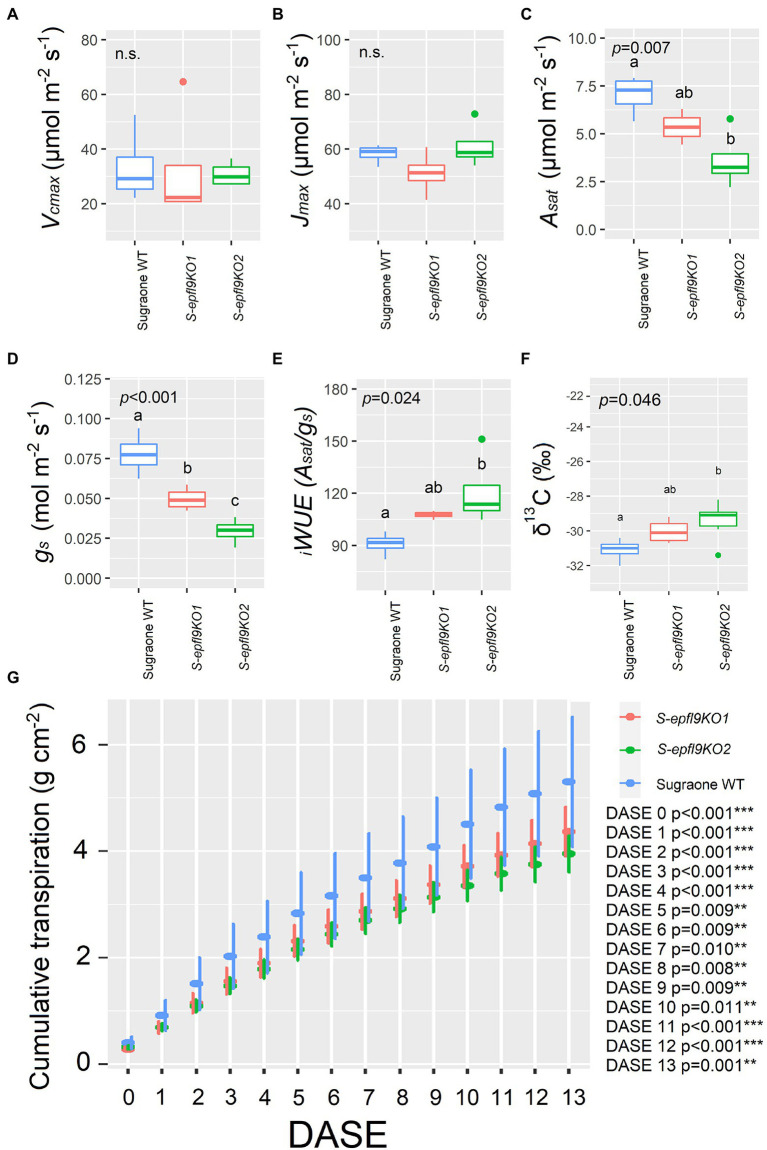
Trait assessment under well-watered (WW) conditions (Experiment 1). **(A)** Maximum velocity of Rubisco carboxylation (*V_cmax_*). **(B)** Maximum electron transport rate for RuBP regeneration (*J_max_*) estimated with *A/C_i_* curves and following curve fitting (Duursma, 2015; Easlon and Bloom, 2014). **(C)** CO_2_ assimilation rate at saturating light (*A_sat_*). **(D)** Stomatal conductance (*g_s_*) extrapolated from *A/C_i_* curves at 400 ppm CO_2_ concentration and 1,500 μmol m^−2^ s^−1^. **(E)** Intrinsic water-use efficiency (*_i_WUE*) calculated as *_i_WUE* = *A_sat_*/*g_s_*. **(F)** Carbon Isotope composition (δ^13^C) analysis. Data were collected on fully expanded leaves of 20 cm tall plants on the twelfth day from the start of the experiment and were elaborated with one-way ANOVA (*n* = 4 in **A**–**E**; *n* = 3–6 in **F**). Whiskers indicate the ranges of the minimum and maximum values and different letters indicate significantly different values according to Fisher’s test. **(G)** Cumulative water loss assessed gravimetrically and normalized for leaf area estimated *via* RGB imaging for a period of 14 days; DASE = Days After Start of the Experiment. Data were means ± standard error of the mean (*n* = 5–6). Data were elaborated with one-way ANOVA for each day (^***^p < 0.001, ^**^p < 0.01, ^*^p < 0.05). When present, different letters indicate significantly different values according to Fisher’s test.

**Figure 6:**
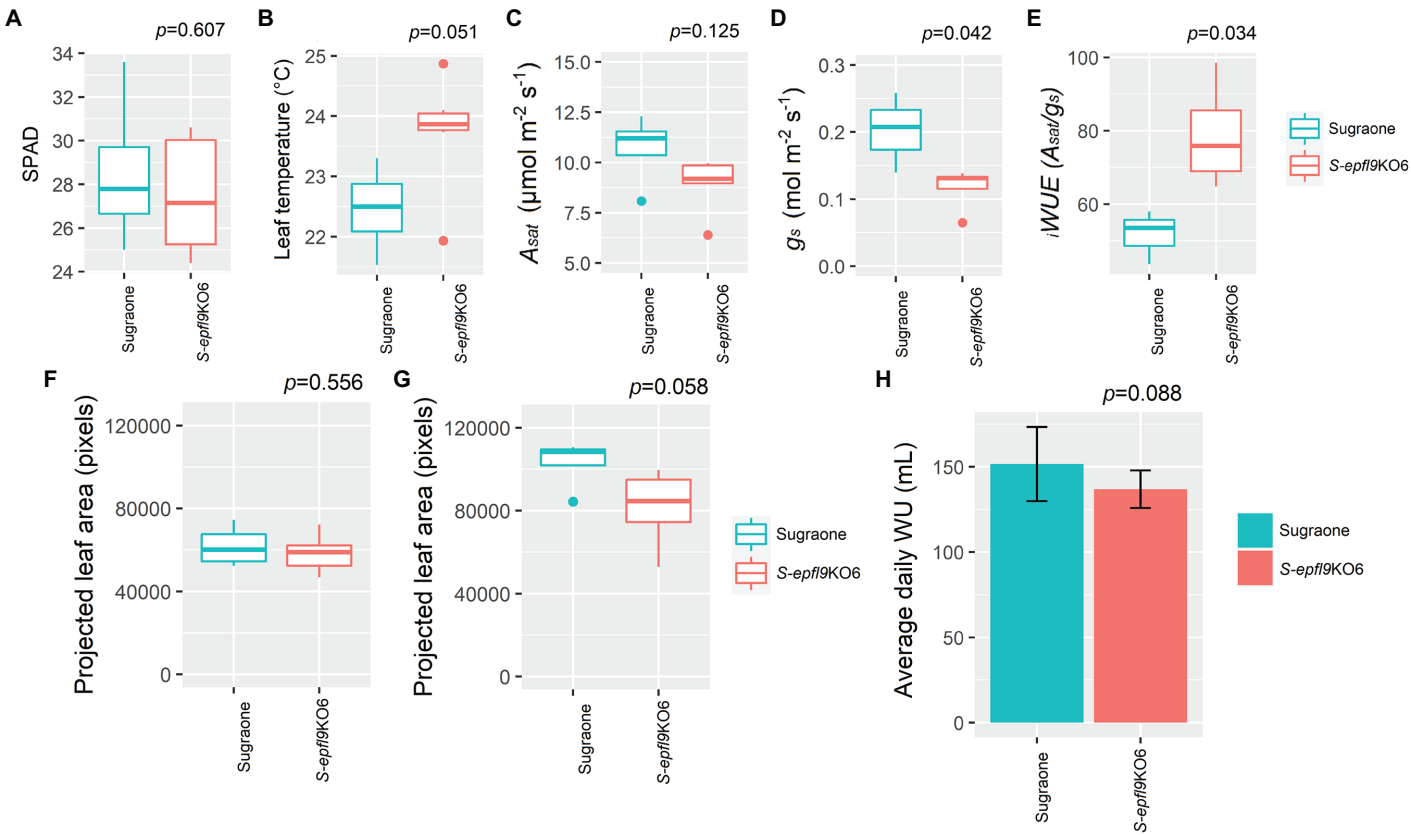
Dynamics of gas exchange, projected leaf area and water-use under well-watered (WW) conditions (Experiment 3) for S-*epfl9*KO6 (*n* = 6) and ‘Sugraone’ WT (*n* = 4). **(A)** SPAD values. **(B)** leaf temperature. **(C)** CO_2_ assimilation rate at saturating light (*A_sat_*). **(D)** stomatal conductance (*g_s_*). **(E)** Intrinsic water-use efficiency (*_i_WUE*) calculated as *_i_WUE* = *A_sat_*/*g_s_*. **(F,G)** projected leaf area (PLA, pixels) collected at day 1 and at day 12, respectively, and **(H)** average daily water-use. For gas exchange measurements, data were collected on fully expanded leaves and were analysed with one-way ANOVA. Whiskers indicate the ranges of the minimum and maximum values.

*A*/*C_i_* response curves (net CO_2_ assimilation rate, *A*, versus calculated substomatal CO_2_ concentration, *C_i_*) were carried out under optimal environmental conditions and saturating light intensity assessed *via* light curves for selected edited lines S-*epfl9*KO1 and S-*epfl9*KO2 in Experiment 1 (Supplementary Figure 8). There were no significant differences for maximum rate of Rubisco-mediated carboxylation (*V_cmax_*) between edited lines and WT control (*p* > 0.05, Figure 5A). Similarly, maximum electron transport rate for RuBP regeneration (*J_max_*) did not vary between edited lines and WT control (*p* > 0.05, Figure 5B). On the contrary, significant reductions in CO_2_ assimilation rate at saturating light (*A_sat_*) were detected for S-*epfl9*KO1 and, in particular, S-*epfl9*KO2 when compared to WT and up to 50% (*p* = 0.007, Figure 5C). S-*epfl9*KO1 and S-*epfl9*KO2 had significantly lower conductance (*g_s_*) than WT (*p* < 0.001) with S-*epfl9*KO2 showing the lowest values (0.030 mol m^−2^ s^−1^ on average, Figure 5D). This led to a significantly higher intrinsic water-use efficiency (*_i_WUE*) for S-*epfl9*KO2 than ‘Sugraone’ WT (*p* = 0.024, Figure 5E). Accordingly, carbon isotope composition (δ^13^C) analysis detected for S-*epfl9*KO2 significant less negative δ^13^C values compared to ‘Sugraone’ WT (*p* = 0.046), indicating a higher *_i_WUE* (Figure 5F). Gravimetric assessments of transpired water normalized for leaf area highlighted significant differences in cumulative transpiration between edited and WT lines. In general, both S-*epfl9*KO1 and S-*epfl9*KO2 used less water throughout a 14 day experimental period, by up to 21%, compared to ‘Sugraone’ WT (Figure 5G). Moreover, to expand our data in well-watered conditions we evaluated the gas-exchange and transpiration performances of an additional line, S-epfl9KO6, maintained in greenhouse for 12 months (Experiment 3). S-*epfl9*KO6 showed similar SPAD values compared to ‘Sugraone’ WT (*p* = 0.607, Figure 6A) and trends were observed for leaf temperature with S-*epfl9*KO6 showing increased leaf temperature (*p* = 0.051, Figure 6B) compared to WT. This increase in leaf temperature was associated with a significant decrease in stomatal conductance (*p* = 0.042, Figure 6D) together with a non-significant difference for *A_sat_* (*p* = 0.125, Figure 6C). This led to a significant increase in *_i_WUE* for S-*epfl9*KO6 compared to control (*p* = 0.034, Figure 6E). No significant differences were observed for projected leaf area (PLA; Figures 6F,G) and water use (WU; Figure 6H) between S-*epfl9*KO6 and ‘Sugraone’ WT although a trend was present for WU (*p* = 0.088).

### The Knock-Out of *VvEPF9-1* May Reduce Impact of Water Stress in Grapevine

**Figure 7:**
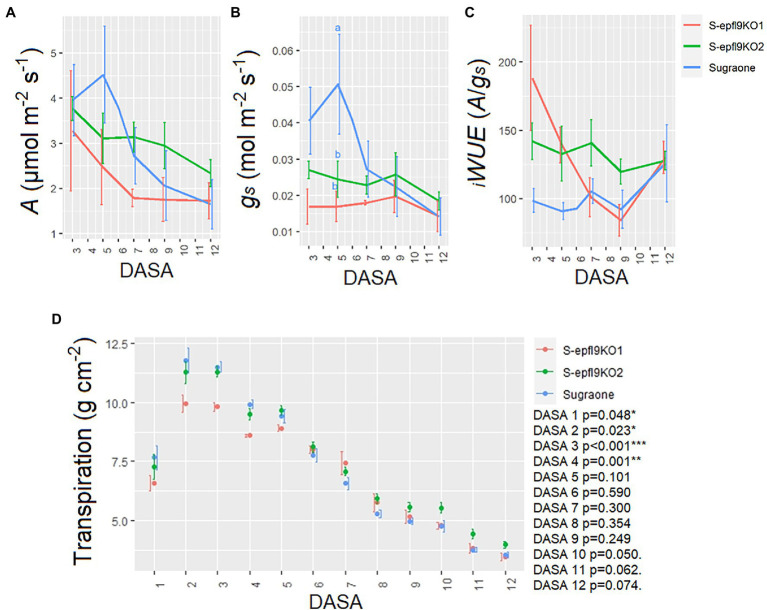
Trait assessment under water stress (WS) conditions. **(A)** *In vivo* CO_2_ assimilation rate at saturating light (*A_sat_*). **(B)** Stomatal conductance (*g_s_*). **(C)** Intrinsic water-use efficiency *_i_WUE* calculated as *_i_WUE* = *A_sat_*/*g_s_*. Data are the means ± standard error of the mean (*n* = 4–6). Data were analysed with one-way ANOVA (value of *p* in the Supplementary Table 5) while different letters indicate significant differences between lines according to Fisher’s test. **(D)** Transpiration assessed gravimetrically and normalized for leaf area estimated *via* RGB imaging. Data are means ± standard error of the mean (*n* = 5–6). Data were analysed with one-way ANOVA (^***^p < 0.001, ^**^p < 0.01, ^*^p < 0.05, *p* < 0.1) for each day. DASA, Days After Stress Application.

*In vivo* gas-exchange measurements at saturating light were carried out throughout the dry down Experiment 2 (Figure 7). ANOVA output for each DASA (Day After Stress Application) is shown in Supplementary Table 5. *In vivo* CO_2_ assimilation rate (*A*) was significantly reduced by water stress (WS) in ‘Sugraone’ WT showing a steeper reduction than knock-out lines, although no significant differences were observed for each day and between lines (Figure 7A). S-*epfl9*KO1 and S-*epfl9*KO2 maintained a lower stomatal conductance (*g_s_*) than ‘Sugraone’ WT (*p* = 0.0276, DASA 5, Figure 7B) but intrinsic water-use efficiency *_i_WUE* resulted not significantly different between the analysed plants (Figure 7C). Transpiration normalized on leaf area was significantly reduced during the WS and for all the lines (Figure 7D). The average fraction of transpirable soil water (FTSW) during the dry down is shown in Supplementary Figure 3. There were significant differences (*p* < 0.05) between S-*epfl9*KO1 and ‘Sugraone’ WT, in particular in the first part of stress application (DASA 1 to 4). Trends (*p* < 0.1) were observed under severe WS (DASA 10 to 12) with S-*epfl9*KO2 having higher transpiration than ‘Sugraone’ WT. Carbon isotope composition (δ^13^C) analysis showed that water stress led to less negative values for all the lines (*p* < 0.001) although no significant differences were observed between edited lines and WT (*p* = 0.186; Supplementary Figure 9).

## Discussion

Crops worldwide will experience warmer conditions in the next decades, followed by limited water availability and increasing atmospheric CO_2_ concentration (McGranahan and Poling, 2018). Alteration of stomatal density and stomatal size through the genetic manipulation of epidermal patterning factors has been shown to be an effective approach to increase drought tolerance and reduce water loss in several species (Bertolino et al., 2019; Buckley et al., 2020). There is a lot of knowledge about *EPF* gene family in *Arabidopsis* and in domesticated grasses but in perennial crops, which present genetic and physiological differences compared to annual species due to ecological and agronomic peculiar features (Lundgren and Marais, 2020), no evidence has been collected on their role. The aim of our study was to shed light for the first time on the genetic basis of stomatal density traits in grapevine, a perennial woody fruit plant with a longer lifespan than previously studied crops (i.e., longer than 30 years).

Water conservation, higher *_i_WUE* and enhanced tolerance to multiple stresses (e.g., drought stress combined with heat stress) were achieved in *Arabidopsis* and grasses overexpressing *EPF1/EPF2* or down-regulating *EPFL9*, due to a reduction in stomatal density (Franks et al., 2015; Hughes et al., 2017; Caine et al., 2019; Dunn et al., 2019; Lu et al., 2019). Between these two reverse genetics approaches, we have chosen the second, relying on the knock-out of *VvEPFL9* by the powerful CRISPR/Cas9 gene editing technology.

In the grapevine genus we found two *AtEPFL9* orthologs, we named *VvEPFL9-1* and *VvEPFL9-2*, identical at 82% in the protein region corresponding to the functional peptide and, respectively, sharing 82 and 95% identity with the same region of AtEPFL9 peptide. So far, two *EPFL9* paralogs have been found in maize and rice (Yin et al., 2017; Hepworth et al., 2018; Lu et al., 2019), showing, respectively, 84 and 73% (*ZmEPFL9-1* and *ZmEPFL9-2*) and 82 and 73% (*OsEPFL9-1* and *OsEPFL9-2*) identities to AtEPFL9 functional peptide. It has been suggested that *EPFL9* paralogs in cereals might be functionally divergent (Lu et al., 2019) but definitive evidence indicating a different function has never been produced. In the study of Lu et al. (2019), the approach used to silence *OsEPF9-1* was RNA interference with a 450 bp-long hairpin RNA, which hardly discriminated between the two variants. In our study, we decided to focus on *VvEPFL9-1*, since at the time the experiment was designed, *VvEPFL9-2* was not anchored to any chromosome in the grapevine reference genome and this uncertainty oriented our choice on *VvEPFL9-1*. According to our data, the knock-out of *VvEPFL9-1* can reduce stomatal density by up to 60%, leading to the hypothesis that *VvEPFL9-1* and *VvEPFL9-2* could be both involved in stomatal induction with a redundant function. A similar approach based on CRISPR/Cas9 technology to knock-out *EPFL9* in rice achieved nearly 90% of stomatal density reduction compared to control by targeting a site on the first exon encoding for the signal peptide and thus not discriminating between *OsEPFL9* paralogs (Yin et al., 2017).

Our study also confirms the crucial role of cysteine residues in the C-terminal functional peptide. This is demonstrated by the lines S-*epfl9*KO5 and S-*epfl9*KO8 in which the loss of the second cysteine (due to a 3 bp-deletion or single base substitution) resulted in a stomatal density reduction similar to the one gained by a full frameshift of the coding sequence. This is consistent with the finding of Ohki et al. (2011) who observed that impairing the formation of a disulphide bond prevented the correct protein folding and function. The design of a sgRNA that directed Cas9 cleavage next to the nucleotide triplet coding for the second cysteine proved to be a good choice for effective 3- and 6- bp deletions. Moreover, our data showed that the retention of almost 50% functional *VvEPFL9-1* in some transgenic lines (S-*epfl9*KO1 and S-*epfl9*KO5) due to a partial editing of the target site, with a substantial maintenance of a WT peptide, still resulted in a significant decrease of SD, suggesting that a threshold amount of peptide may be required for EPFL9-1 to be functionally effective.

Reduction in stomatal density following *VvEPFL9-1* knock-out was significant, although partially compensated by an increase in stomatal size (SS, inferred by pore length measurements). The negative yet non-linear association between SD and SS has been frequently reported in many species (Franks and Beerling, 2009) and often linked to an improved economy of epidermal space allocation with the combination of low SD and high SS as a preferable strategy when low stomatal conductance is required (Doheny-Adams et al., 2012; Lawson and McElwain, 2016). In our work, however, the reduction in SD was accompanied by only a partial compensation for SS.

Stomata are the main drivers of transpiration but at the same time are pivotal for CO_2_ uptake for mesophyll photosynthesis (Lawson and Blatt, 2014). For instance, in barley and wheat, a reduction in SD by 50% compared to WT led to a significant reduction in carbon assimilation (*A_sat_*) and conductance (*g_s_*) and to an enhanced water use efficiency (*_i_WUE*) under optimal growth conditions (Hughes et al., 2017; Dunn et al., 2019). Similarly, in two-months-old ‘Sugraone’ at well-watered conditions (Experiment 1), we found that a 60% reduction in SD led to a reduced *A_sat_* for the edited lines compared to the WT. Additionally, the reduction in *g_s_* was even greater, leading to a higher value of *_i_WUE* (i.e., *A_sat_*/ *g_s_*) in edited versus WT lines. Moreover, the reduction in *A_sat_* was not concomitant to reductions in Rubisco velocity (*V_cmax_*) or to impairment in electron transport chain (*J_max_*) suggesting that the knock-out of *VvEPFL9-1* did not affect the photosynthetic machinery, at least at the conditions applied in this work. In an additional experiment (Experiment 3) carried out under well-watered conditions in a phenotyping platform on older plants than those used in experiment 1, *iWUE* confirmed to be significantly improved in the edited line (S-*epfl9*KO6) compared to ‘Sugraone’ WT while transpiration (WU) performances were not significantly different. The main sources of variation between the two experiments were plant age and environmental conditions. In Experiment 3 plants were older than in Experiment 1 (12- vs. 2-Months-old) and regarding light conditions, Experiment 1 was carried out under the natural fluctuating light of a greenhouse, while in Experiment 3 plants were subject to a steady-state light pattern. Our results suggest that canopy structure (over-saturation of apical leaves and basal leaves under the sub-saturating light intensities of the greenhouse) may play a role in defining the effectiveness of a reduced stomatal density phenotype. Furthermore, the conditions of dynamic light intensity such as those present in the greenhouse, may have contributed to accentuate the water saving behavior of the lines with lower stomatal density. Indeed, reducing stomatal density can limit stomatal clustering (Harrison et al., 2020) and therefore increase stomatal responsiveness to environmental cues (Faralli et al., 2021). Important differences for g_s_ were also observed between Experiments 1 and 3, suggesting that plant age and pot-effect significantly influences operating g_s_, although the g_s_ values are inside the ranges shown by Lavoie-Lamoureux et al. (2017) for pot-grown grapevine. *Vitis vinifera* genotypes with reduced SD and, in turn, limited *A_sat_* and greater *_i_WUE*, may be desirable to improve plant water conservation and to delay sugar accumulation under current and future climatic scenarios (Kuhn et al., 2014; Arrizabalaga-Arriazu et al., 2021). Sugars and organic acids along with various secondary metabolites (e.g., tannins, flavonols, anthocyanins, aroma compounds) are determinants of grape berry quality and their accumulation during berry ripening is the result of the interaction between genotype and environment, a relationship made vulnerable by climate change (Bobeica et al., 2015; Rienth et al., 2021). It is known that grapevine physiology will be impacted by elevated carbon dioxide, increasing temperatures, and extreme heat events during the growing season (De Cortázar-Atauri et al., 2017; Delrot et al., 2020). In particular, high temperature and increasing CO_2_ levels are already affecting viticulture (Cook and Wolkovich, 2016; Mosedale et al., 2016; Edwards et al., 2017; Droulia and Charalampopoulos, 2021) with an evident shift towards an earlier onset of phenological stages (Edwards et al., 2017; Alikadic et al., 2019) and accelerated berry ripening (Jones et al., 2005; Parker et al., 2020; Rienth et al., 2021). High temperatures and water stress slow down vine metabolism resulting in a lower accumulation of polyphenols and aromatic compounds in the berries (Tomasi et al., 2011; Jones, 2013; Pons et al., 2017; Venios et al., 2020). Thus, one of the consequences of a compressed phenology may be an earlier sugar accumulation in the berries that leads to anticipated harvest dates when the secondary metabolites content is sub-optimal (Palliotti et al., 2014; Edwards et al., 2017). Although currently several agronomic approaches of source-limitation (i.e., pre-flowering leaf removal, shading nets, anti-transpirant application, etc.) have been set up to delay sugar accumulation in ripening grapes in the field (Palliotti et al., 2014; Prats-Llinàs et al., 2020), stomatal manipulation may be a favorable genetic strategy for the future, that deserves to be further explored also under combined environmental stress and in field trials. In our study, we further applied a water stress experiment to test if and how a reduced stomatal density can affect plant behavior in drought conditions. During a progressive reduction in soil water availability, significant differences in transpiration rate were observed in edited lines compared to WT only under moderate water stress (i.e., DASA 3 and 4). Yet, under severe water stress (e.g., DASA 10–12), some trends (*p* < 0.1) were observed in edited lines showing higher transpiration rate followed by *A_sat_* and *g_s_* maintenance. Notably, the reduction in *g_s_* and *A_sat_* during the dry-down was evident for WT plants (*p* < 0.001) while this was not significant for edited lines. This conservative behavior induced by reduced SD has been previously associated with a longer period of transpiration maintenance during drought, leading to a prolonged carbon assimilation respect to WT (Caine et al., 2019). In rice, lines overexpressing the *OsEPF1* gene had higher yield than WT when water-stressed at flowering stage (Caine et al., 2019) confirming that water conservation during key-stages of yield formation may be desirable for yield maintenance (Faralli et al., 2019). In addition, limiting plant transpiration could be an advantage for irrigated vineyards in terms of a reduction in water input demand (Keller et al., 2016). In view of an increase in the number of grapevine growing regions where water resources will become limited (Schultz, 2000; Santillán et al., 2020), genotypes with reduced stomatal density will require less units of irrigation water for cultivation area, thus increasing crop water productivity for farmers (Scholasch and Rienth, 2019).

## Conclusion

To our knowledge, this is the first study describing the function of *VvEPFL9-1* in a perennial fruit crop as well as the physiological advantages of *epfl9-1* knocked-out phenotype under different availability of soil water. In grapevine, reducing stomatal density *via VvEPFL9-1* loss of function can induce water conservation and increase *_i_WUE*, although an impact of photosynthetic CO_2_ absorbance (*A_sat_*) was observed in some edited lines. While in several crops, reduced photosynthetic CO_2_ uptake can decrease yield and biomass, we speculate that reduced *A_sat_* and increased *_i_WUE* may be a favorable combination of physiological attributes in grapevine, especially under future climate change scenario. However, at this stage further trials in the field under standard management conditions are required as well as additional evaluations regarding the potential effects of reduced stomatal density under natural environmental fluctuations. To conclude, this work reinforces the concept that stomatal anatomical features constitute a promising target for designing climate change-resilient crops (Franks et al., 2015; Hughes et al., 2017; Bertolino et al., 2019; Caine et al., 2019; Dunn et al., 2019; Lu et al., 2019; Buckley et al., 2020) and provides evidence of this in grapevine, the most economically important fruit crop globally.

## Data Availability Statement

The datasets presented in this study can be found in online repositories. The names of the repository/repositories and accession number(s) can be found in the article/Supplementary Material. The original contributions presented in the study are publicly available. This data can be found here: NCBI Sequence Read Archive, BioProject accession number: PRJNA820619.

## Author Contributions

MC performed plant transformation experiments, plant molecular analysis, phenotyping, statistical analysis, and wrote the paper. MF contributed to the dry-down experiment design, carried out Experiment 3, supervised physiological analysis and statistical elaboration of the data, and wrote the paper. JL carried out alignments and phylogenetic tree and revised the manuscript. LB performed carbon isotope composition analysis and revised the manuscript. SP performed the analysis for the T-DNA integration point determination. CV, MM, WO, and AR supervised and revised the manuscript. LDC conceived the project, designed vectors for gene editing, performed paralogs analysis, transformation experiments, plant molecular characterization, took care of the plants in greenhouse, and wrote the paper. All authors contributed to the article and approved the submitted version.

## Funding

This research was funded by the Autonomous Province of Trento (Italy) in the framework of the Fondazione Edmund Mach (FEM) International PhD initiative and by the ERDF 2014–2020 Program of the Autonomous Province of Trento with EU co-financing (Fruitomics, CUP number: C49H18000000001).

## Conflict of Interest

The authors declare that the research was conducted in the absence of any commercial or financial relationships that could be construed as a potential conflict of interest.

## Publisher’s Note

All claims expressed in this article are solely those of the authors and do not necessarily represent those of their affiliated organizations, or those of the publisher, the editors and the reviewers. Any product that may be evaluated in this article, or claim that may be made by its manufacturer, is not guaranteed or endorsed by the publisher.

## Footnotes

^1^ blast.ncbi.nlm.nih.gov

^2^ https://www.dna-cloning.com/

^3^ http://crispr.hzau.edu.cn/cgi-bin/CRISPR2/CRISPR

^4^ https://crispresso.pinellolab.partners.org/submission

^5^ blast.ncbi.nlm.nih.gov

^6^ https://integrape.eu/resources/genes-genomes/genome-accessions/

^7^ https://integrape.eu/resources/genes-genomes/genome-accessions/

^8^ http://www.cbs.dtu.dk/services/SignalP/

^9^ http://plants.ensembl.org/index.html
