# Supplementary material for: VvEPFL9-1 Knock-Out via CRISPR/Cas9 Reduces Stomatal Density in Grapevine
Source: Front Plant Sci. 2022 May 17;13:878001. doi: 10.3389/fpls.2022.878001 (PMC9152544; doi:10.3389/fpls.2022.878001)
Supplement: Supplementary file 1 [file Table_1.DOCX]

| Species | Cultivar | Reference | | Web site |
| --- | --- | --- | --- | --- |
| *Vitis arizonica* | - | ^1^ | http://www.grapegenomics.com/pages/Vari/ | |
| *Vitis riparia* | Gloire de Montpellier | ^2^ | https://www.ncbi.nlm.nih.gov/assembly/GCF_004353265.1 | |
| *Vitis sylvestris* | DVIT3351.27 | ^1^ | http://www.grapegenomics.com/pages/VvSyl/ | |
|  | DVIT3603.07 | ^1^ | http://www.grapegenomics.com/pages/VvSyl/ | |
|  | DVIT3603.16 | ^1^ | http://www.grapegenomics.com/pages/VvSyl/ | |
|  | O34-16 | ^1^ | http://www.grapegenomics.com/pages/VvSyl/ | |
| *Vitis vinifera* | Black Corinth Seeded | ^1^ | http://www.grapegenomics.com/pages/VvBlaCori/ | |
|  | Black Corinth Seedless | ^1^ | http://www.grapegenomics.com/pages/VvBlaCori/ | |
|  | Cabernet Sauvignon | ^3^ | http://www.grapegenomics.com/pages/VvCabSauv/ | |
|  | Carménère | ^4^ | http://www.grapegenomics.com/pages/VvCar/ | |
|  | Merlot | ^1^ | http://www.grapegenomics.com/pages/VvMerl/ | |
|  | Riesling | ^5^ | http://www.grapegenomics.com/pages/VvRies/ | |
|  | Pinot Noir ENTAV115 | ^6^ | https://www.ncbi.nlm.nih.gov/bioproject/PRJEA18357 | |
|  | Pinot Noir (PN40024) | ^7,8^ | https://urgi.versailles.inra.fr/Species/Vitis/Data-Sequences/Genome-sequences | |

**Supplementary Table 1.** Publicly available genomic databases of different *Vitis Spp.* and *Vitis vinifera* varieties.

1. Massonnet, M. *et al.* The genetic basis of sex determination in grapes. *Nat. Commun.* **11**, 1–12 (2020).

2. Girollet, N. *et al.* De novo phased assembly of the *Vitis riparia* grape genome. *Sci. Data* **6**, 1–8 (2019).

3. Chin, C. S. *et al.* Phased diploid genome assembly with single-molecule real-time sequencing. *Nat. Methods* **13**, 1050–1054 (2016).

4. Minio, A., Massonnet, M., Figueroa-Balderas, R., Castro, A. & Cantu, D. Diploid genome assembly of the wine grape carménère. *G3 Genes, Genomes, Genet.* **9**, 1331–1337 (2019).

5. Zou, C. *et al.* Multiple independent recombinations led to hermaphroditism in grapevine. *Proc. Natl. Acad. Sci. U. S. A.* **118**, (2021).

6. Velasco, R. *et al.* A high quality draft consensus sequence of the genome of a heterozygous grapevine variety. *PLoS One* **2**, (2007).

7. Vitulo, N. *et al.* A deep survey of alternative splicing in grape reveals changes in the splicing machinery related to tissue, stress condition and genotype. *BMC Plant Biol.* **14**, 20–30 (2014).

8. Canaguier, A. *et al.* A new version of the grapevine reference genome assembly (12X.v2) and of its annotation (VCost.v3). *Genomics Data* **14**, 56–62 (2017).
