# Supplementary material for: VvEPFL9-1 Knock-Out via CRISPR/Cas9 Reduces Stomatal Density in Grapevine
Source: Front Plant Sci. 2022 May 17;13:878001. doi: 10.3389/fpls.2022.878001 (PMC9152544; doi:10.3389/fpls.2022.878001)
Supplement: Supplementary file 2 [file Table_2.DOCX]

| Primer name | Primer sequence | PCR product lenght | Application |
| --- | --- | --- | --- |
| VvEPFL9-1_fw | 5′-GGGACTGCAACTCATTCAGAACT-3′ | 450 bp | 1. Sequencing of the VvEPFL9 genes in genotypes of interest 2. check of the editing in the target site (on/off target) |
| VvEPFL9-1_rv | 5′-TCTCCTACATCCCACATGCATCT-3’ |  |  |
| VvEPFL9-2_fw | 5′-GGGAACAAGTAGTATCTATGCCT-3′ | 308 bp |  |
| VvEPFL9-2_rv | 5′- TGATCAACACAACACTGAGCCT-3′ |  |  |
| SpCas9_Fw | 5′-CTTCAGAAAGGACTTCCAATTC-3′ | 693 bp | Screening of the transgenic plants |
| SpCas9_Rv | 5′-ATGATCAAGTCCTTCTTCACTT-3′ |  |  |
| VvChiRT_fw | 5′-GAGGCTGGGGATGAGAAAATTG-3′ | 75 bp | CN quantification by Real-time PCR |
| VvChiRT_rv | 5′-CCCATCTCTCCTTCAACCACCT-3′ |  |  |
| VvChiRT_Probe | FAM-5′-AAGCTGAGAAGG TTGCTCCGGT-3′-TAMRA |  |  |
| SpCas9RT_fw | 5′-TACGCTGACCTTTTCTTGG-3’ | 87 bp |  |
| SpCas9RT_rv | 5′-CTTGGTGATCTCAGTGTTCA-3′ |  |  |
| SpCas9RT_Probe | FAM-5′-CCTCTCCGACGCTATTCTGCTCTCC-3′ |  |  |
| 35S_plasmid_rv^a^ | 5′-GGGCAATGGAATCCGAGGA-3′ | ^b^ | Integration site validation |
| KO1_Chr18_fw | 5′-CCGGGCAAACTCTCCTTCTA-3′ | ^b^ |  |
| KO2_Chr1_fw | 5′-ATATCCGCAGACACAGACGT-3′ | ^b^ |  |
| KO3_Chr13_fw | 5′-CCTAAGATGAGTGCGAGGGT-3′ | ^b^ |  |
| KO6_Chr4_fw | 5′-CACTTCATTAGGTGCTTGTCA-3′ | ^b^ |  |
| KO7_Chr3_fw | 5′-GCTAAGACGAGGATTGGAATGG-3′ | ^b^ |  |

**Supplementary Table 5.** Primers and probes used in the PCR reactions for different applications. ^a^ 35S_plasmid_rv was used in combination with all the fw primers used for the “integration site validation”. ^b^ PCR product length is dependent from the random T-DNA insertion in the plant genome
