# Supplementary material for: VvEPFL9-1 Knock-Out via CRISPR/Cas9 Reduces Stomatal Density in Grapevine
Source: Front Plant Sci. 2022 May 17;13:878001. doi: 10.3389/fpls.2022.878001 (PMC9152544; doi:10.3389/fpls.2022.878001)
Supplement: Supplementary file 3 [file Table_3.DOCX]

**Supplementary Table 3**. Sanger sequencing of the *VvEPFL9-1* and *VvEPFL9-2* PCR amplified fragments from genomic DNA extracted from leaves of *in-vitro* grapevine varieties. Primer used in the PCR were reported in Supplemental Table S5 (VvEPFL9-1_fw; VvEPFL9-1_rv; VvEPFL9-2_fw; VvEPFL9-2_rv). In red the coding sequence for the functional domain of the protein. The position of the SNPs is assigned considering the coding region in red; upstream of that region, SNP position is identified as the bases of distance from the beginning of the red region preceded by a minus (-). Sequencing data were deposited in the public repository “NCBI Sequence Read Archive”, BioProject accession number: PRJNA820619 (release date 2022-03-28).

| **Grapevine variety** | **Sequence ID** | **Sanger sequenced region** | **Allelic polymorphism** |
| --- | --- | --- | --- |
| **Cabernet Sauvignon** | ***>VviCabSau_EPFL9-1*** | TTCATATCCATCACAAAGAATAGTATCAGTTCAGGTGAGAAATGCAAACATTAATGAAGATCAAAACCAGTCCCATATTCCTTGATTTTTTTCAATAAGTATGTGCTTATATTTAGGGTTCTTGGGTGCTAGAACAATGGAGATGGAGGATGGAGAAAATGGGGTTCAAGAAGAGAGATGATAGGGTCTACAGCCCCAACATGCTCATACAATGAATGCAAAGGGTGCAAGTTCAAGTGCAGAGCAGAGCAGATTCCTGTGGATGGTAATGACCCAATTCACAGTGCCTATCACTACAAGTGTATGTGCCATAGGTAATTCAATTAGTGGCTCCTTTTTTTTTTTTTGGGTTCTTTTTTGGAAAGTTT | 2 SNPs:  G + T (position -97);  T + A (position 25) |
|  | ***>VviCabSau_EPFL9-2*** | TGGGAACAAGTAGTATCTATGCCTGAAAATTTGTTCAATTATACTCGAGTATACTTACTTTTGACCATTTTTGAGCCTGCAGGGCAGTGGTGAACAATGGATGAATAGGAATTCAAGGAGACTGATGATTGGATCCACCCGGCCAACCTGCACTTACAATGAATGTAGAGGGTGTAAGTACAAGTGCAGAGCTGAGCAAGTACCGGTCGAGGGGAACGACCCGATTAATAGCGCATACCACTATAGATGCGTTTGTCATAGGTAAAGATGAACCAAAGTTAAGGCTAAGGCTCAGTGTTGTGTTGATCAA | 1 SNP:  G + A (position -45) |
| **Sugraone** | ***>VviSugra_EPFL9-1*** | TTCATATCCATCACAAAGAGTAGTATCAGTTCAGGTGAGAAATGCAAACATTAATGAAGATCAAAACCAGTCCCATATTCCTTGATTTTTTTCAATAAGTATGTGCTTATATTTAGGGTTCTTGGGTGCTAGAACAATGGAGATGGAGGATGGAGAAAATGGGTTTCAAGAAGAGAGATGATAGGGTCTACAGCCCCAACATGCACATACAATGAATGCAAAGGGTGCAAGTTCAAGTGCAGAGCAGAGCAGATTCCTGTGGATGGTAATGACCCAATTCACAGTGCCTATCACTACAAGTGTATGTGCCATAGGTAATTCAATTAGTGGCTCCTTTTTTTTTTTTTGGGTTCTTTTTTGGTAAGTTTT | 2 SNPs:  G + T (position -97); G + T (position -17) |
|  | ***>VviSugra_EPFL9-2*** | TGGGAACAAGTAGTATCTATGCCTGAAAATTTGTTCAATTATACTCGAGTATACTTACTTTTGACCATTTTTGAGCCTGCAGGGCAGTGGTGAACAATGGATGAATAGGAATTCAAGGAGACTGATGATTGGATCCACCCGGCCAACCTGCACTTACAATGAATGTAGAGGGTGTAAGTACAAGTGCAGAGCTGAGCAAGTACCGGTCGAGGGGAACGACCCGATTAATAGCGCATACCACTATAGATGCGTTTGTCATAGGTAAAGATGAACCAAAGTTAAGGCTAAGGCTCAGTGTTGTGTTGATCAA | 1 SNP:  G + A (position -45) |
| **Pinot Noir (PN40024)** | ***>VviPN40024_EPFL9-1*** | TTCATATCCATCACAAAGAGTAGTATCAGTTCAGGTGAGAAATGCAAACATTAATGAAGATCAAAACCAGTCCCATATTCCTTTATTTTTTTCAATAAGTATGTGCTTATATTTAGGGTTCTTGGGTGCTAGAACAATGGAGATGGAGGATGGAGAAAATGGGGTTCAAGAAGAGAGATGATAGGGTCTACAGCCCCAACATGCACATACAATGAATGCAAAGGGTGCAAGTTCAAGTGCAGAGCAGAGCAGATTCCTGTGGATGGTAATGACCCAATTCACAGTGCCTATCACTACAAGTGTATGTGCCATAGGTAATTCAATTAGTGGCTCCTTTTTTTTTTT | No SNP |
|  | ***>VviPN40024_EPFL9-2*** | TGGGAACAAGTAGTATCTATGCCTGAAAATTTGTTCAATTATACTCGAGTATACTTACTTTTGACCATTTTTGAGCCTGCAGAGCAGTGGTGAACAATGGATGAATAGGAATTCAAGGAGACTGATGATTGGATCCACCCGGCCAACCTGCACTTACAATGAATGTAGAGGGTGTAAGTACAAGTGCAGAGCTGAGCAAGTACCGGTCGAGGGGAACGACCCGATTAATAGCGCATACCACTATAGATGCGTTTGTCATAGGTAAAGATGAACCAAAGATAAGGCTAAGGCTCAGTGTTGTGATGATCA | No SNP |
| **Pinot Noir (ENTAV115)** | ***>VviPN-ENTAV115_EPFL9-1*** | TTCATATCCATCACAAAGAGTAGTATCAGTTCAGGTGAGAAATGCAAACATTAATGAAGATCAAAACCAGTCCCATATTCCTTTATTTTTTTCAATAAGTATGTGCTTATATTTAGGGTTCTTGGGTGCTAGAACAATGGAGATGGAGGATGGAGAAAATGGGGTTCAAGAAGAGAGATGATAGGGTCTACAGCCCCAACATGCACATACAATGAATGCAAAGGGTGCAAGTTCAAGTGCAGAGCAGAGCAGATTCCTGTGGATGGTAATGACCCAATTCACAGTGCCTATCACTACAAGTGTATGTGCCATAGGTAATTCAATTAGTGGCTCCTTTTTTTTTTT | No SNP |
|  | ***>Vvi-ENTAV115_EPFL9-2*** | TGGGAACAAGTAGTATCTATGCCTGAAAATTTGTTCAATTATACTCGAGTATACTTACTTTTGACCATTTTTGAGCCTGCAGAGCAGTGGTGAACAATGGATGAATAGGAATTCAAGGAGACTGATGATTGGATCCACCCGGCCAACCTGCACTTACAATGAATGTAGAGGGTGTAAGTACAAGTGCAGAGCTGAGCAAGTACCGGTCGAGGGGAACGACCCGATTAATAGCGCATACCACTATAGATGCGTTTGTCATAGGTAAAGATGAACCAAAGATAAGGCTAAGGCTCAGTGTTGTATTGATCAA | No SNP |
| **Merlot** | ***>VviMer_EPFL9-1*** | TTCATATCCATCACAAAGAGTAGTATCAGTTCAGGTGAGAAATGCAAACATTAATGAAGATCAAAACCAGTCCCATATTCCTTGATTTTTTTCAATAAGTATGTGCTTATATTTAGGGTTCTTGGGTGCTAGAACAATGGAGATGGAGGATGGAGAAAATGGGGTTCAAGAAGAGAGATGATAGGGTCTACAGCCCCAACATGCACATACAATGAATGCAAAGGGTGCAAGTTCAAGTGCAGAGCAGAGCAGATTCCTGTGGATGGTAATGACCCAATTCACAGTGCCTATCACTACAAGTGTATGTGCCATAGGTAATTCAATTAGTGGCTCCTTTTTTTTTTTT | 1 SNP:  G + T (position -97) |
|  | ***>VviMer_EPFL9-2*** | TGGGAACAAGTAGTATCTATGCCTGAAAATTTGTTCAATTATACTCGAGTATACTTACTTTTGACCATTTTTGAGCCTGCAGGGCAGTGGTGAACAATGGATGAATAGGAATTCAAGGAGACTGATGATTGGATCCACCCGGCCAACCTGCACTTACAATGAATGTAGAGGGTGTAAGTACAAGTGCAGAGCTGAGCAAGTACCGGTCGAGGGGAACGACCCGATTAATAGCGCATACCACTATAGATGCGTTTGTCATAGGTAAAGATGAACCAAAGTTAAGGCTAAGGCTCAGTGTTGTATTGATCAA | 1 SNP:  G + A (position -45) |
| ***Vitis riparia* ‘Glorie de Montpellier’** | ***>Vri _EPFL9-1*** | TTCATATCCATCACAAAGAGTAGTATCAGTTCAGGTGAGAAATGCAAACATTAATGAAGATCAAAACCAGTCCCATATTCCTTTATTTTTTTCAATAAGTATGTGCTTATATTTAGGGTTCTTGGGTGCTAGAACAATGGAGATGGAGGATGGAGAAAATGGGGTTCAAGAAGAGAGATGATAGGGTCTACAGCCCCAACATGCACATACAATGAATGCAAAGGGTGCAAGTTCAAGTGCAGAGCAGAGCAGATTCCTGTGGATGGTAATGACCCAATTCACAGTGCCTATCACTACAAGTGTATGTGCCATAGGTAATTCAATGAGTGGCTCCTTTTTTTTTTTTTTTT | No SNP |
|  | ***>Vri _EPFL9-2*** | TGGGAACAAGTAGTATCTATGCCTGAAAATTTGTTCAATTATACTCGAGTATACTTACTTTTGACCATTTTTGAGCCTGCAGAGCAGTGGTGAACAATGGATGAATAGGAATTCAAGGAGACTGATGATTGGATCCACCCGGCCAACCTGCACTTACAATGAATGTAGAGGGTGTAAGTACAAGTGCAGAGCTGAGCAAGTACCGGTCGAGGGGAACGACCCGATTAATAGCGCATACCACTATAGATGCGTTTGTCATAGGTAAAGATGAACCAAAGTTAAGGCTAAGGCTCAGTGTT | No SNP |
| **Chardonnay** | ***>VviCha_EPFL9-1*** | TTCATATCCATCACAAAGAGTAGTATCAGTTCAGGTGAGAAATGCAAACATTAATGAAGATCAAAACCAGTCCCATATTCCTTTATTTTTTTCAATAAGTATGTGCTTATATTTAGGGTTCTTGGGTGCTAGAACAATGGAGATGGAGGATGGAGAAAATGGGGTTCAAGAAGAGAGATGATAGGGTCTACAGCCCCAACATGCACATACAATGAATGCAAAGGGTGCAAGTTCAAGTGCAGAGCAGAGCAGATTCCTGTGGATGGTAATGACCCAATTCACAGTGCCTATCACTACAAGTGTATGTGCCATAGGTAATTCAATTAGTGGCTCCTTTTTTTTTTTTT | 1 SNP:  G + T (position -97) |
|  | ***>VviCha_EPFL9-2*** | TGGGAACAAGTAGTATCTATGCCTGAAAATTTGTTCAATTATACTCGAGTATACTTACTTTTGACCATTTTTGAGCCTGCAGAGCAGTGGTGAACAATGGATGAATAGGAATTCAAGGAGACTGATGATTGGATCCACCCGGCCAACCTGCACTTACAATGAATGTAGAGGGTGTAAGTACAAGTGCAGAGCTGAGCAAGTACCGGTCGAGGGGAACGACCCGATTAATAGCGCATACCACTATAGATGCGTTTGTCATAGGTAAAGATGAACCAAAGTTAAGGCTAAGGCTCAGTGTTGTGTTGATCAA | No SNP |
| **Syrah** | ***>VviSyr_EPFL9-1*** | TTCATATCCATCACAAAGAGTAGTATCAGTTCAGGTGAGAAATGCAAACATTAATGAAGATCAAAACCAGTCCCATATTCCTTGATTTTTTTCAATAAGTATGTGCTTATATTTAGGGTTCTTGGGTGCTAGAACAATGGAGATGGAGGATGGAGAAAATGGGGTTCAAGAAGAGAGATGATAGGGTCTACAGCCCCAACATGCACATACAATGAATGCAAAGGGTGCAAGTTCAAGTGCAGAGCAGAGCAGATTCCTGTGGATGGTAATGACCCAATTCACAGTGCCTATCACTACAAGTGTATGTGCCATAGGTAATTCAATTAGTGGCTCCTTTTTTTTTTTT | 1 SNP:  G + T (position -97) |
|  | ***>VviSyr_EPFL9-2*** | TGGGAACAAGTAGTATCTATGCCTGAAAATTTGTTCAATTATACTCGAGTATACTTACTTTTGACCATTTTTGAGCCTGCAGGGCAGTGGTGAACAATGGATGAATAGGAATTCAAGGAGACTGATGATTGGATCCACCCGGCCAACCTGCACTTACAATGAATGTAGAGGGTGTAAGTACAAGTGCAGAGCTGAGCAAGTACCGGTCGAGGGGAACGACCCGATTAATAGCGCATACCACTATAGATGCGTTTGTCATAGGTAAAGATGAACCAAAGTTAAGGCTAAGGCTCAGTGTTGTGTTGATCAA | 1 SNP:  G + A (position -45) |
| **Touriga Nacional** | ***>VviTouNat_EPFL9-1*** | TTCATATCCATCACAAAGAGTAGTATCAGTTCAGGTGAGAAATGCAAACATTAATGAAGATCAAAACCAGTCCCATATTCCTTGATTTTTTTCAATAAGTATGTGCTTATATTTAGGGTTCTTGGGTGCTAGAACAATGGAGATGGAGGATGGAGAAAATGGGGTTCAAGAAGAGAGATGATAGGGTCTACAGCCCCAACATGCACATACAATGAATGCAAAGGGTGCAAGTTCAAGTGCAGAGCAGAGCAGATTCCTGTGGATGGTAATGACCCAATTCACAGTGCCTATCACTACAAGTGTATGTGCCATAGGTAATTCAATTAGTGGCTCCTTTTTTTTTTT | 1 SNP:  G + T (position -97) |
|  | ***>VviTouNat_EPFL9-2*** | GGGGAACAAGTAGTATCTATGCCTGAAAATTTGTTCAATTATACTCGAGTATACTTACTTTTGACCATTTTTGAGCCTGCAGAGCAGTGGTGAACAATGGATGAATAGGAATTCAAGGAGACTGATGATTGGATCCACCCGGCCAACCTGCACTTACAATGAATGTAGAGGGTGTAAGTACAAGTGCAGAGCTGAGCAAGTACCGGTCGAGGGGAACGACCCGATTAATAGCGCATACCACTATAGATGCGTTTGTCATAGGTAAAGATGAACCAAAGTTAAGGCTAAGGCTCAGTGTTGTGTTGATCAA |  |
