# Supplementary material for: VvEPFL9-1 Knock-Out via CRISPR/Cas9 Reduces Stomatal Density in Grapevine
Source: Front Plant Sci. 2022 May 17;13:878001. doi: 10.3389/fpls.2022.878001 (PMC9152544; doi:10.3389/fpls.2022.878001)
Supplement: Supplementary file 4 [file Table_4.DOCX]

**Supplementary Table 4.** Outputs of CRISPResso2 software for the analysis of CRISPR/*Cas9* genome editing outcomes from Illumina sequencing data for all the edited lines obtained. For each transgenic line, the distribution of identified alleles around the predicted Cas9 cleavage position is reported (column 2). The 20bp-target site in the exon3 of VvEPFL9-1 (i.e. GCACATACAATGAATGCAAA) is indicated with a grey horizontal bar on each plot. Nucleotides are indicated by unique colors (A = green; C = red; G = yellow; T = purple). Substitutions are shown in bold font. Red rectangles highlight inserted sequences. Horizontal dashed lines indicate deleted sequences. The vertical dashed line indicates the predicted cleavage site. For the 9 selected lines reported in Fig. 2, mutated alleles were translated in protein with the software EMBO Transeq (<https://www.ebi.ac.uk/Tools/st/emboss_transeq/>) and the kind of mutation classified as (i) frameshift mutation with premature stop codons (F-S with SC); (ii) frameshift mutation without stop codons (F-S without SC); (iii) loss of the second Cysteine of the 6 –Cys-array (Loss of Cys). The presence of a non-mutated allele resulted in a % of WT (wild type) peptide. Alleles with a reduced number of reads (i.e. values of percentages that are below 1) were not considered (most of them showed some polymorphisms far away from the Cas9 cleavage site that are probably due to technical errors of the PCR reaction or of the sequencing procedure). According to the plot, the sum of the percentages for each kind of mutation does not reach 100% (column 5), so, for clarity of presentation and to normalize the outputs among lines, percentages were proportionally increased to reach a total of 100% (column 6, P%= proportionally increased percentages).

| **Lines ID** | **Allele plot: identified alleles around the cleavage site** | **Prediction of protein mutations in the C-terminal peptide** | | **Mutation %** | |
| --- | --- | --- | --- | --- | --- |
| S-*epfl9KO1* | 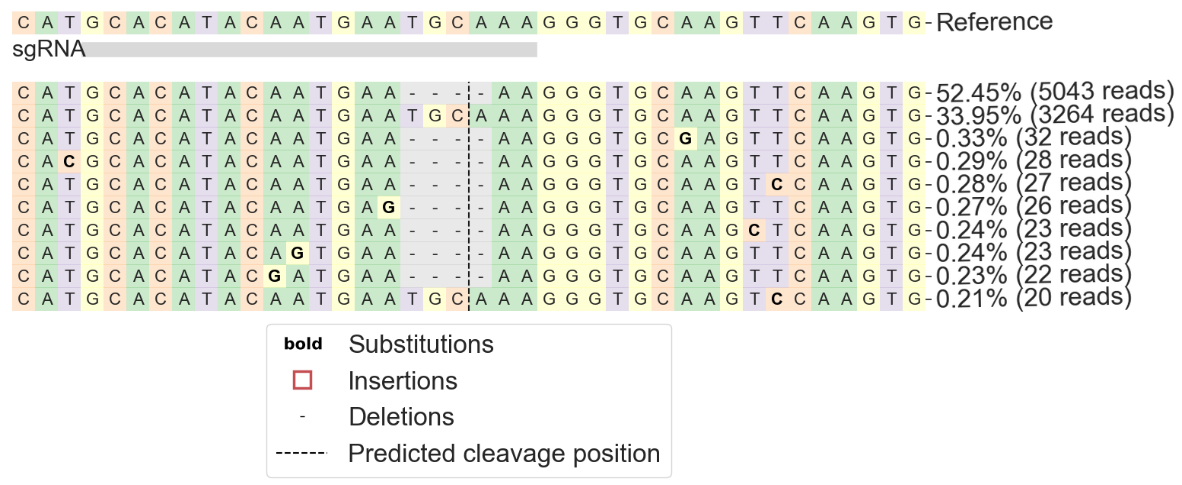 | MIGSTAPT**C**TYNE**C**KG**C**KFK**C**RAEQIPVDGNDPIHSAYHYK**C**M**C**HR  MIGSTAPT**C**TYNE**KGASSSAEQSRFLWMVMTQFTVPITTSVCAIGX**  MIGSTAPT**C**TYNE**C**KG**C**KFK**C**RAEQIPVDGNDPIHSAYHYK**C**M**C**HR | Reference peptide  FS without SC  WT | %  52.45%  33.95% | P%  60,7%  39,3% |
| S-*epfl9KO2* | 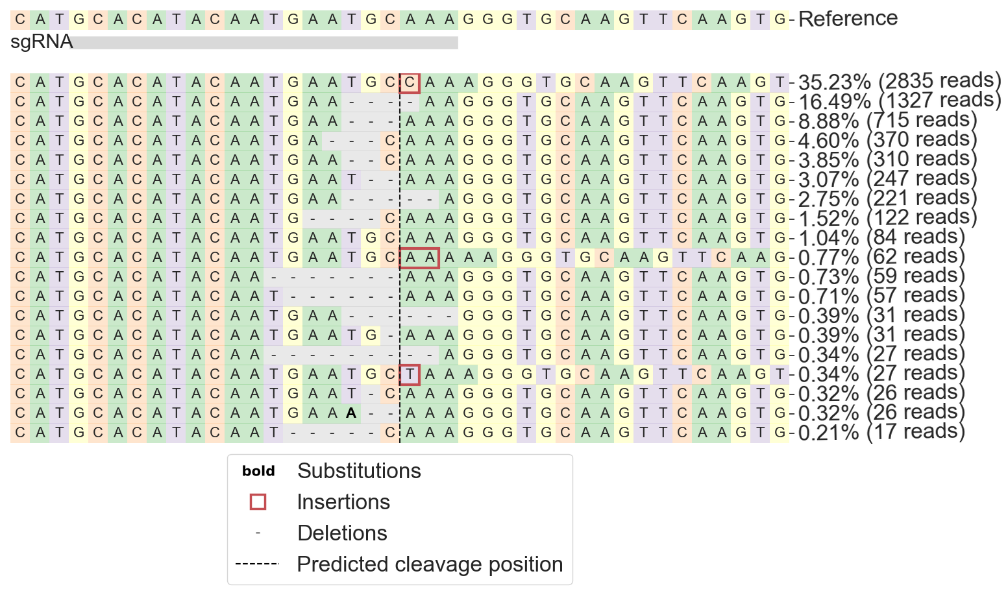 | MIGSTAPT**C**TYNE**C**KG**C**KFK**C**RAEQIPVDGNDPIHSAYHYK**C**M**C**HR  MIGSTAPT**C**TYNE**KGASSSAEQSRFLWMVMTQFTVPITTSVCAIGX**  MIGSTAPT**C**TYNE**CQRVQVQVQSRADSCGW****  MIGSTAPT**C**TYNE**K**G**C**KFK**C**RAEQIPVDGNDPIHSAYHYK**C**M**C**HR  MIGSTAPT**C**TYN**D**KG**C**KFK**C**RAEQIPVDGNDPIHSAYHYK**C**M**C**HR  MIGSTAPT**C**TYNE**QRVQVQVQSRADSCGW****  MIGSTAPT**C**TYNE*****  MIGSTAPT**C**TYNE**RVQVQVQSRADSCGW****  MIGSTAPT**C**TYN**AKGASSSAEQSRFLWMVMTQFTVPITTSVCAIGX**  MIGSTAPT**C**TYNE**C**KG**C**KFK**C**RAEQIPVDGNDPIHSAYHYK**C**M**C**HR | Reference peptide  FS without SC  FS with SC  Loss of Cys  Loss of Cys  FS with SC  FS with SC  FS with SC  FS without SC  WT | %  35.23%  16.49%  8.88%  4.60%  3.85%  3.07%  2.75%  1.52%  1.04% | P%  45.50%  21.30%  11.47%  5.94%  4.97%  3.96%  3.55%  1.96%  1.34% |
| S-*epfl9KO3* | 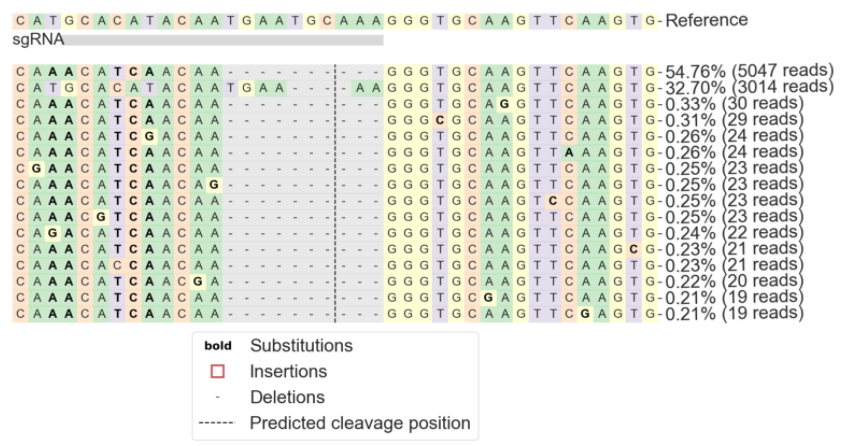 | MIGSTAPT**C**TYNE**C**KG**C**KFK**C**RAEQIPVDGNDPIHSAYHYK**C**M**C**HR  MIGSTAP**NINKGASSSAEQSRFLWMVMTQFTVPITTSVCAIGX**  MIGSTAPT**C**TYNE**CQRVQVQVQSRADSCGW**** | Reference peptide  FS without SC  FS with SC | %  54.76%  32.70% | P%  62.61%  37.39% |
| S-*epfl9KO4* | 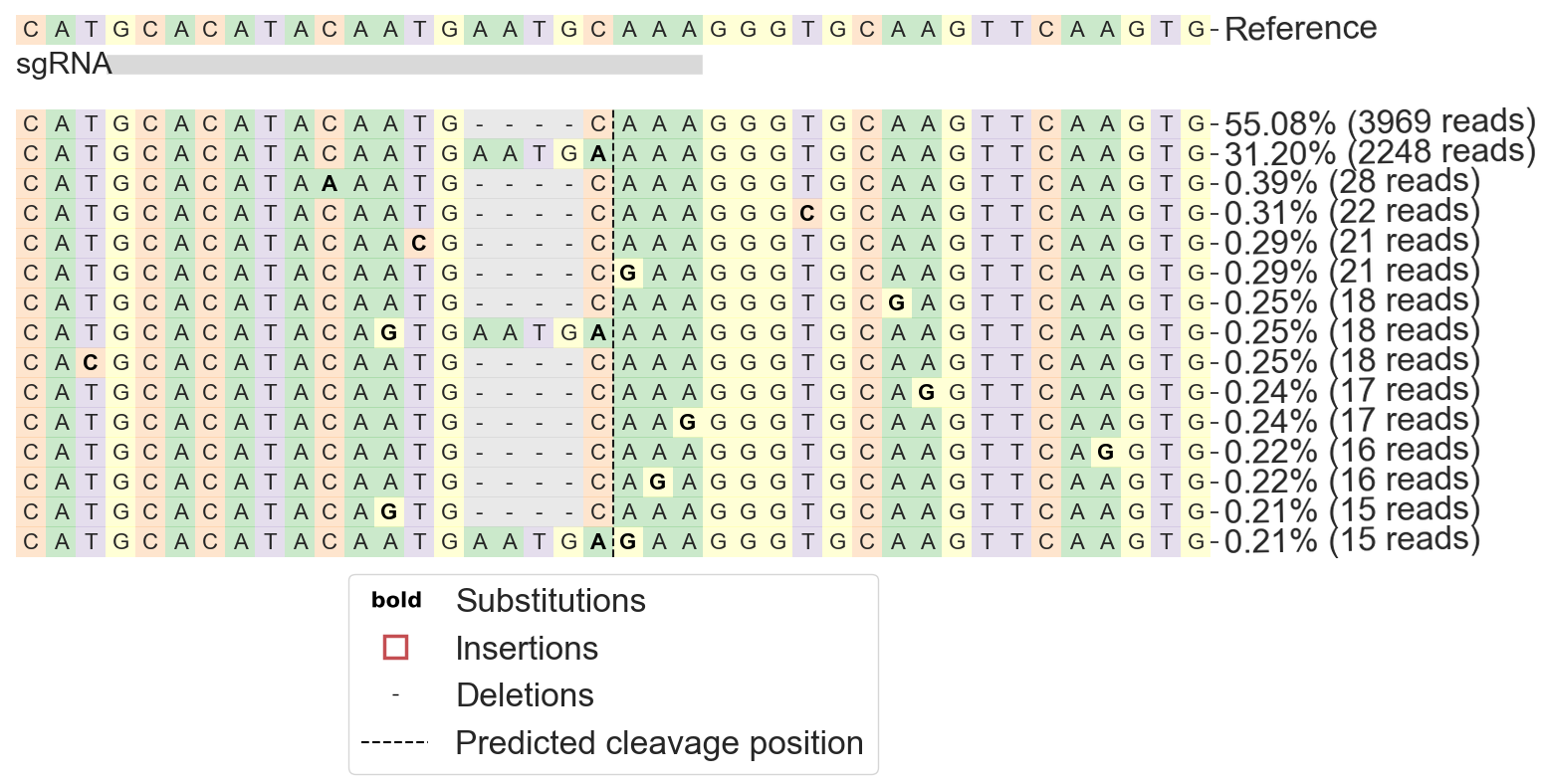 | MIGSTAPT**C**TYNE**C**KG**C**KFK**C**RAEQIPVDGNDPIHSAYHYK**C**M**C**HR  MIGSTAPTCTYN**AKGASSSAEQSRFLWMVMTQFTVPITTSVCAIGX**  MIGSTAPTCTYNE*****KGCKFKCRAEQIPVDGNDPIHSAYHYKCMCHR | Reference peptide  FS without SC  Loss of Cys *****  ***** replaced by SC | %  55.08%  31.20% | P%  63.84%  36.16% |
| S-*epfl9KO5* | 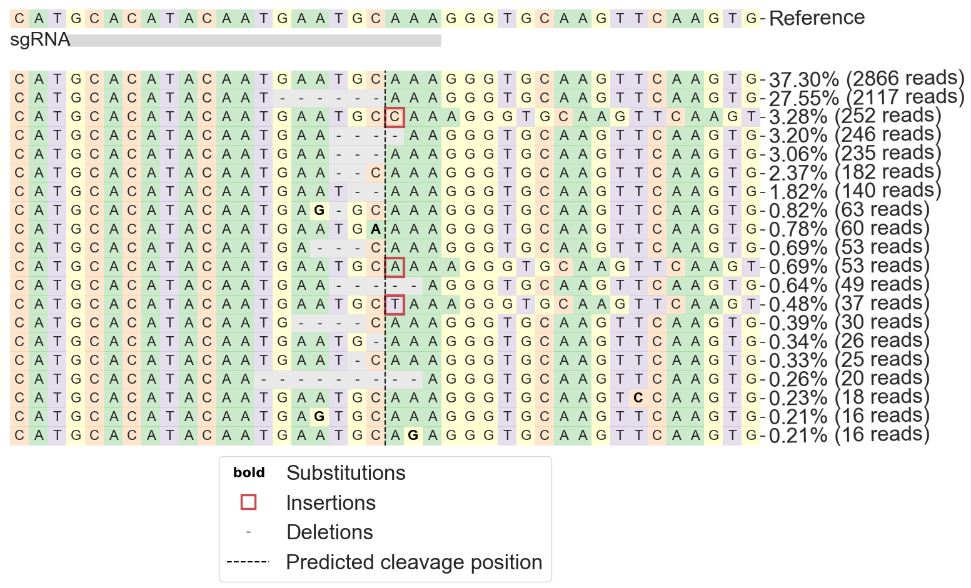 | MIGSTAPT**C**TYNE**C**KG**C**KFK**C**RAEQIPVDGNDPIHSAYHYK**C**M**C**HR  MIGSTAPT**C**TYNE**C**KG**C**KFK**C**RAEQIPVDGNDPIHSAYHYK**C**M**C**HR  MIGSTAPT**C**TYN**K**G**C**KFK**C**RAEQIPVDGNDPIHSAYHYK**C**M**C**HR  MIGSTAPT**C**TYNE**KGASSSAEQSRFLWMVMTQFTVPITTSVCAIGX**  MIGSTAPT**C**TYNE**CQRVQVQVQSRADSCGW****  MIGSTAPT**C**TYNE**K**G**C**KFK**C**RAEQIPVDGNDPIHSAYHYK**C**M**C**HR  MIGSTAPT**C**TYNE**QRVQVQVQSRADSCGW****  MIGSTAPT**C**TYNE***** | Reference peptide  WT  Loss of Cys  FS without SC  FS with SC  Loss of Cys  FS with SC  FS with SC | %  37.30%  27.55%  3.28%  3.20%  3.06%  2.37%  1.82% | P%  47.47%  35.06%  4.17%  4.07%  3.89%  3.02%  2.32% |
| S-*epfl9KO6* | 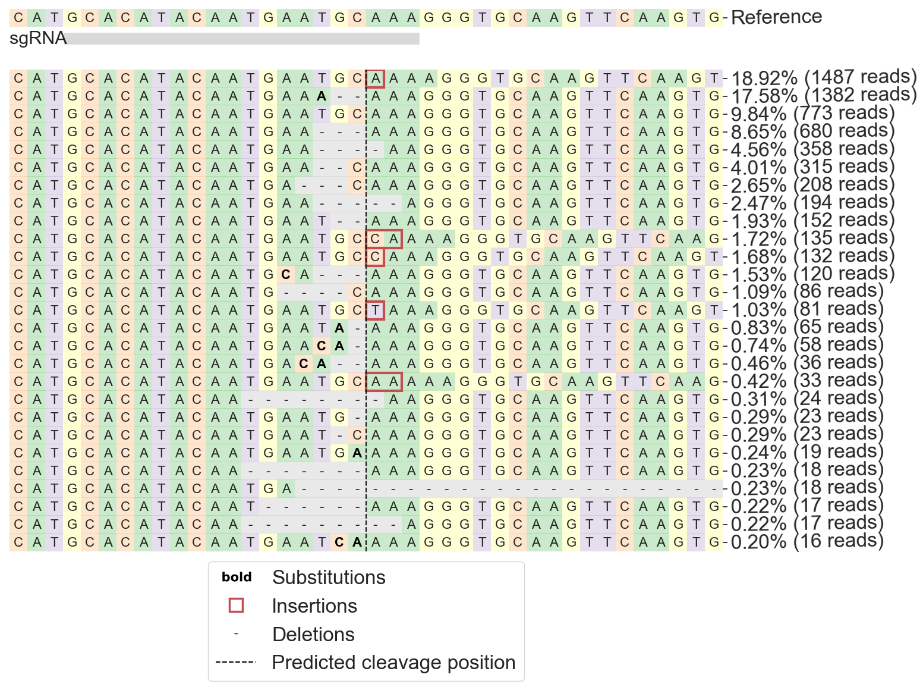 | MIGSTAPT**C**TYNE**C**KG**C**KFK**C**RAEQIPVDGNDPIHSAYHYK**C**M**C**HR  MIGSTAPT**C**TYNE**C**K**RVQVQVQSRADSCGW****  MIGSTAPT**C**TYNE**KRVQVQVQSRADSCGW****  MIGSTAPT**C**TYNE**C**KG**C**KFK**C**RAEQIPVDGNDPIHSAYHYK**C**M**C**HR  MIGSTAPT**C**TYNE**K**G**C**KFK**C**RAEQIPVDGNDPIHSAYHYK**C**M**C**HR  MIGSTAPT**C**TYNE**CQRVQVQVQSRADSCGW****  MIGSTAPT**C**TYNE**QRVQVQVQSRADSCGW****  MIGSTAPT**C**TYN**D**KG**C**KFK**C**RAEQIPVDGNDPIHSAYHYK**C**M**C**HR  MIGSTAPT**C**TYNE**RVQVQVQSRADSCGW****  MIGSTAPT**C**TYNE*****  MIGSTAPT**C**TYNE**CQKGASSSAEQSRFLWMVMTQFTVPITTSVCAIGX**  MIGSTAPT**C**TYN**EKGASSSAEQSRFLWMVMTQFTVPITTSVCAIGX**  MIGSTAPT**C**TYN**A**KG**C**KFK**C**RAEQIPVDGNDPIHSAYHYK**C**M**C**HR  MIGSTAPT**C**TYN**AKGASSSAEQSRFLWMVMTQFTVPITTSVCAIGX**  MIGSTAPT**C**TYNE**C*** | Reference peptide  FS with SC  FS with SC  WT  Loss of Cys  FS with SC  FS with SC  Loss of Cys  FS with SC  FS with SC  FS without SC  FS without SC  Loss of Cys  FS without SC  FS with SC | %  18.92%  17.58%  9.84%  8.65%  4.56%  4.01%  2.65%  2.47%  1.93%  1.72%  1.68%  1.53%  1.09%  1.03% | P%  24.36%  22.64%  12.67%  11.14%  5.87%  5.16%  3.41%  3.18%  2.49%  2.21%  2.16%  1.97%  1.40%  1.33% |
| S-*epfl9KO7* | 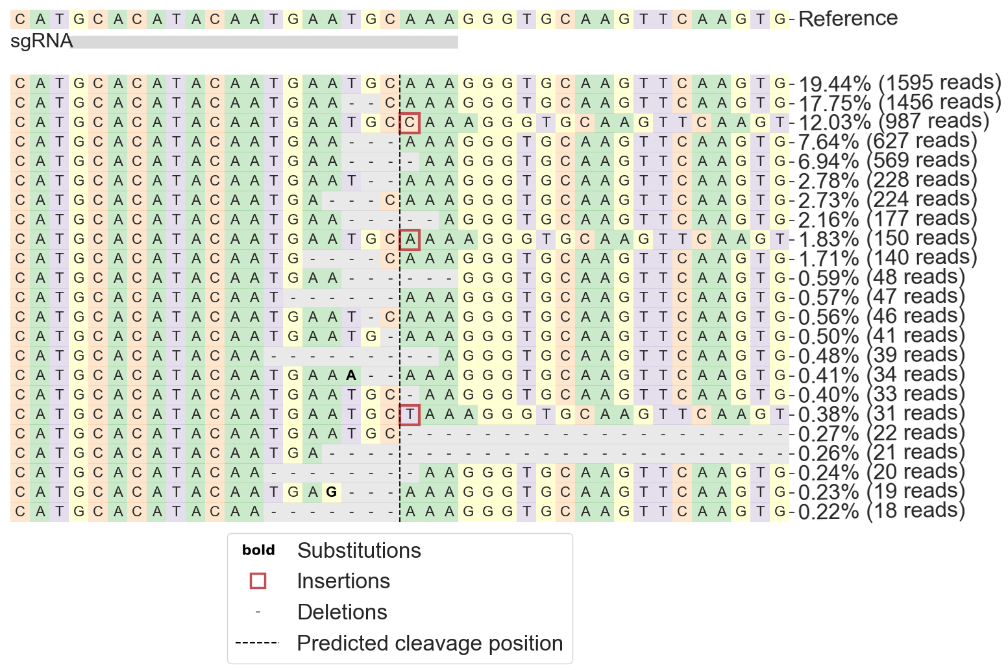 | MIGSTAPT**C**TYNE**C**KG**C**KFK**C**RAEQIPVDGNDPIHSAYHYK**C**M**C**HR  MIGSTAPT**C**TYNE**C**KG**C**KFK**C**RAEQIPVDGNDPIHSAYHYK**C**M**C**HR  MIGSTAPT**C**TYNE**QRVQVQVQSRADSCGW****  MIGSTAPT**C**TYNE**KGASSSAEQSRFLWMVMTQFTVPITTSVCAIGX**  MIGSTAPT**C**TYNE**K**GCKFKCRAEQIPVDGNDPIHSAYHYKCMCHR  MIGSTAPT**C**TYNE**CQRVQVQVQSRADSCGW****  MIGSTAPT**C**TYNE*****  MIGSTAPT**C**TYN**DK**GCKFKCRAEQIPVDGNDPIHSAYHYKCMCHR  MIGSTAPT**C**TYNE**RVQVQVQSRADSCGW****  MIGSTAPT**C**TYNE**C**K**RVQVQVQSRADSCGW****  MIGSTAPT**C**TYN**AKGASSSAEQSRFLWMVMTQFTVPITTSVCAIGX** | Reference peptide  WT  FS with SC  FS without SC  Loss of Cys  FS with SC  FS with SC  Loss of Cys  FS with SC  FS with SC  FS without SC | %  19.44%  17.75%  12.03%  7.64%  4.56%  2.78%  2.73%  2.16%  1.83%  1.71% | P%  25.92%  23.66%  16.04%  10.19%  9.25%  3.71%  3.64%  2.88%  2.44%  2.28% |
| S-*epfl9KO8* | 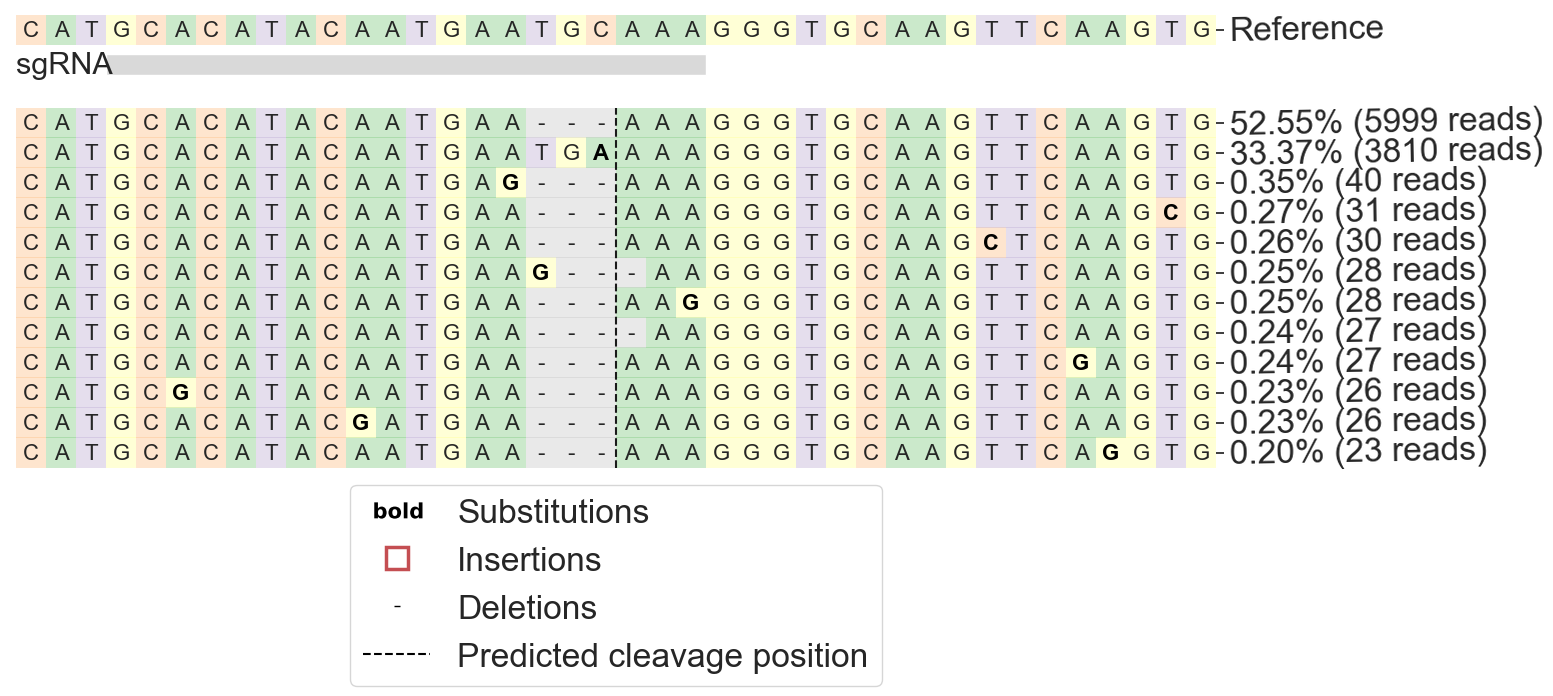 | MIGSTAPT**C**TYNE**C**KG**C**KFK**C**RAEQIPVDGNDPIHSAYHYK**C**M**C**HR  MIGSTAPT**C**TYNE**K**G**C**KFK**C**RAEQIPVDGNDPIHSAYHYK**C**M**C**HR  MIGSTAPT**C**TYNE*****KGCKFKCRAEQIPVDGNDPIHSAYHYKCMCHR | Reference peptide  Loss of Cys  Loss of Cys *****  *****replaced by SC | %  52.55%  33.37% | P%  61.16%  38.84% |
| S-*epfl9KO9* | 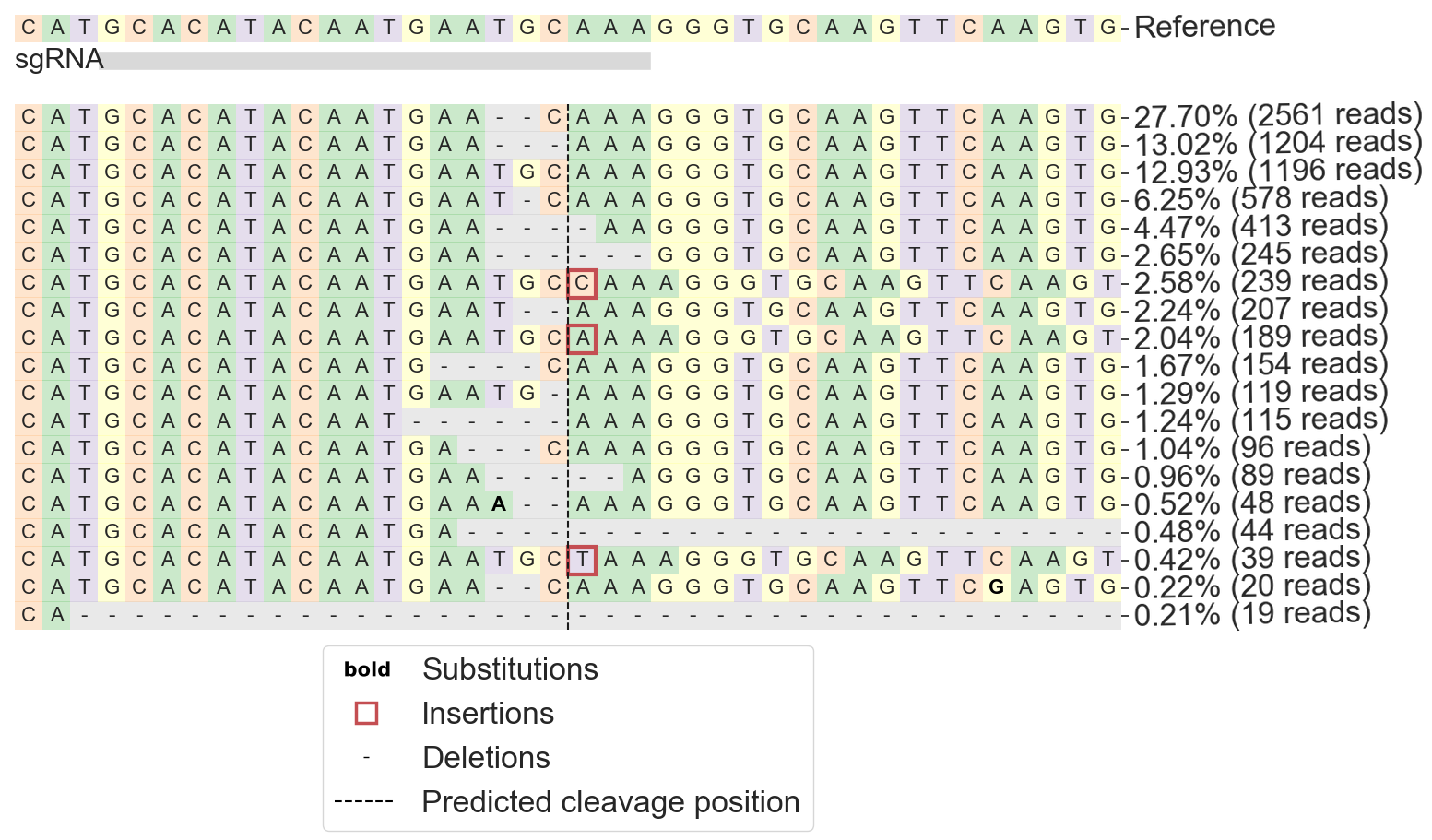 | MIGSTAPT**C**TYNE**C**KG**C**KFK**C**RAEQIPVDGNDPIHSAYHYK**C**M**C**HR  MIGSTAPT**C**TYNEQ**RVQVQVQSRADSCGW****  MIGSTAPT**C**TYNE**K**G**C**KFK**C**RAEQIPVDGNDPIHSAYHYK**C**M**C**HR  MIGSTAPT**C**TYNE**C**KG**C**KFK**C**RAEQIPVDGNDPIHSAYHYK**C**M**C**HR  MIGSTAPT**C**TYNE**SKGASSSAEQSRFLWMVMTQFTVPITTSVCAIGX**  MIGSTAPT**C**TYNE**CQRVQVQVQSRADSCGW****  MIGSTAPT**C**TYNE**GC**KFK**C**RAEQIPVDGNDPIHSAYHYK**C**M**C**HR  MIGSTAPT**C**TYNE**KGASSSAEQSRFLWMVMTQFTVPITTSVCAIGX**  MIGSTAPT**C**TYNE*  MIGSTAPT**C**TYNE**C**K**RVQVQVQSRADSCGW****  MIGSTAPT**C**TYN**AKGASSSAEQSRFLWMVMTQFTVPITTSVCAIGX**  MIGSTAPT**C**TYNE*****  MIGSTAPT**C**TYN**K**G**C**KFK**C**RAEQIPVDGNDPIHSAYHYK**C**M**C**HR  MIGSTAPT**C**TYN**D**KG**C**KFK**C**RAEQIPVDGNDPIHSAYHYK**C**M**C**HR | Reference peptide  FS with SC  Loss of Cys  WT  FS without SC  FS with SC  Loss of Cys  FS without SC  FS with SC  FS with SC  FS without SC  FS with SC  Loss of Cys  Loss of Cys | %  27,70%  13,02%  12,93%  6,25%  4,47%  2,65%  2,58%  2,24%  2,04%  1,67%  1,29%  1,24%  1,04% | P%  35,01%  16,46%  16,34%  7,90%  5,65%  3,35%  3,26%  2,83%  2,58%  2,11%  1,63%  1,57%  1,31% |
| S-*epfl9KO10* | 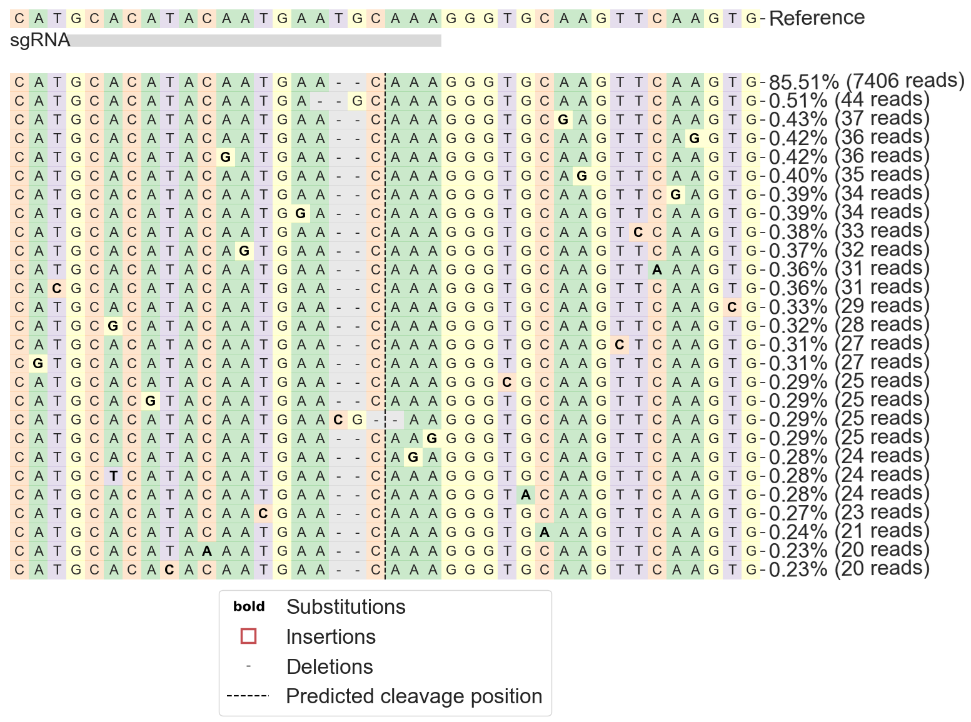 |  |  |  |  |
| S-*epfl9KO11* | 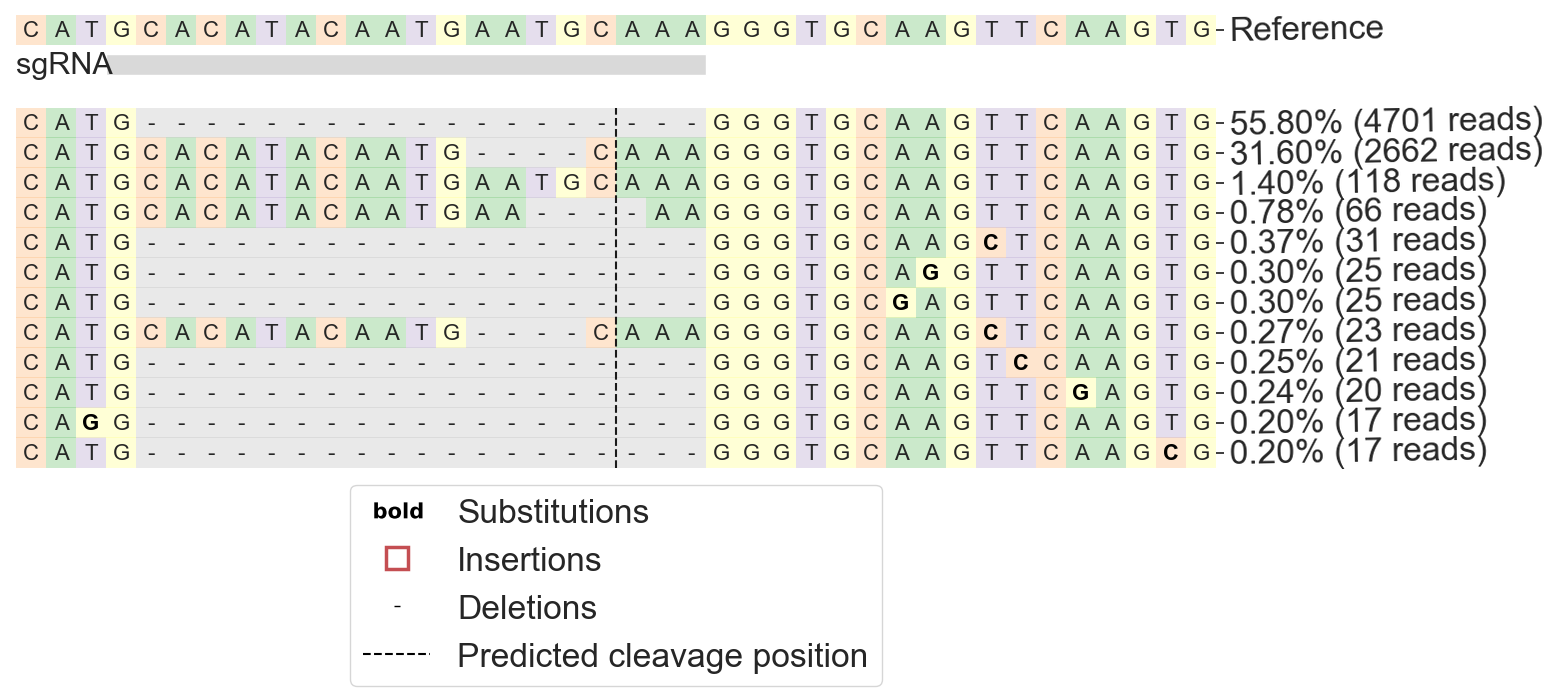 |  |  |  |  |
| S-*epfl9KO12* | 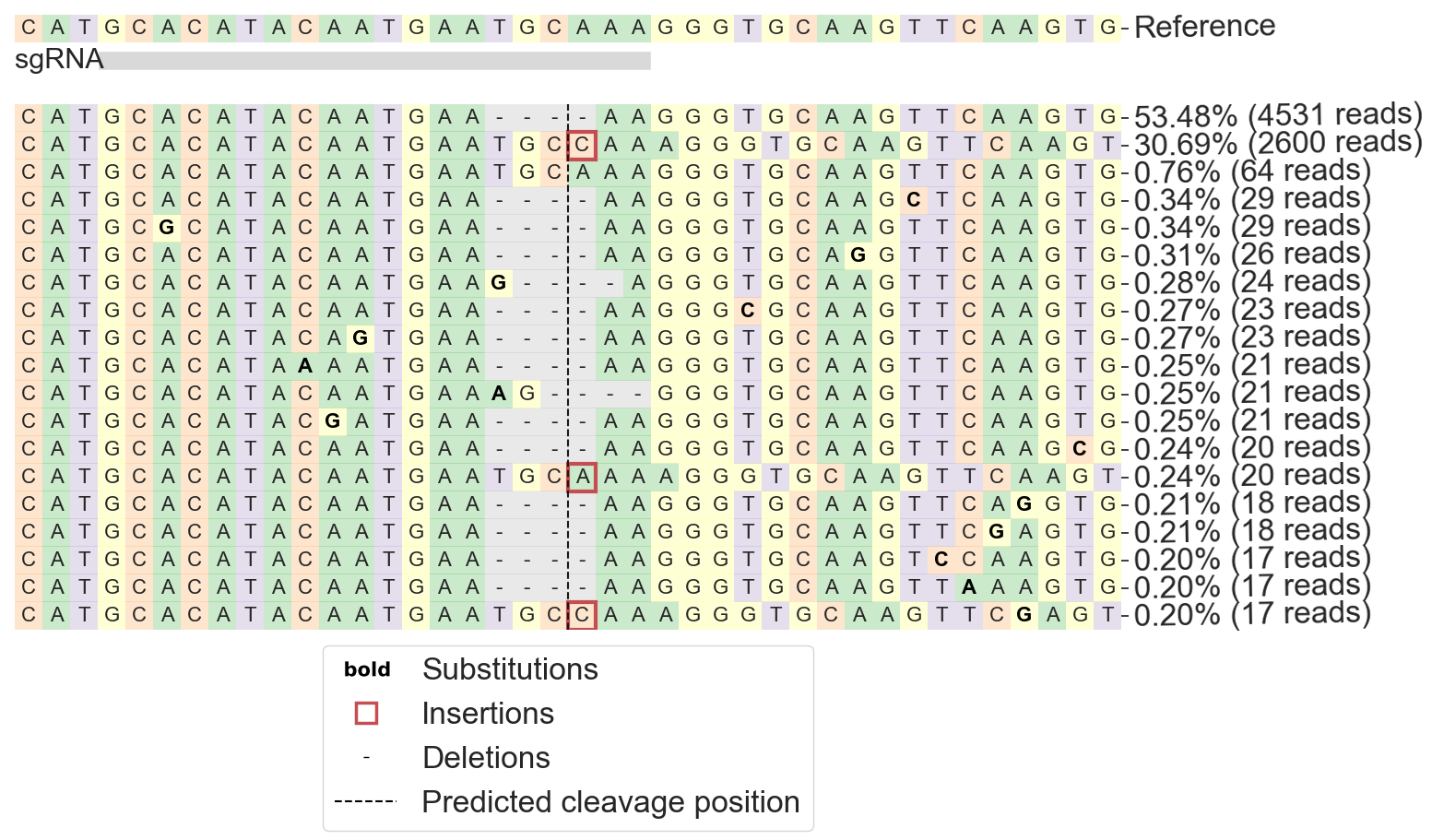 |  |  |  |  |
| S-*epfl9KO13* | 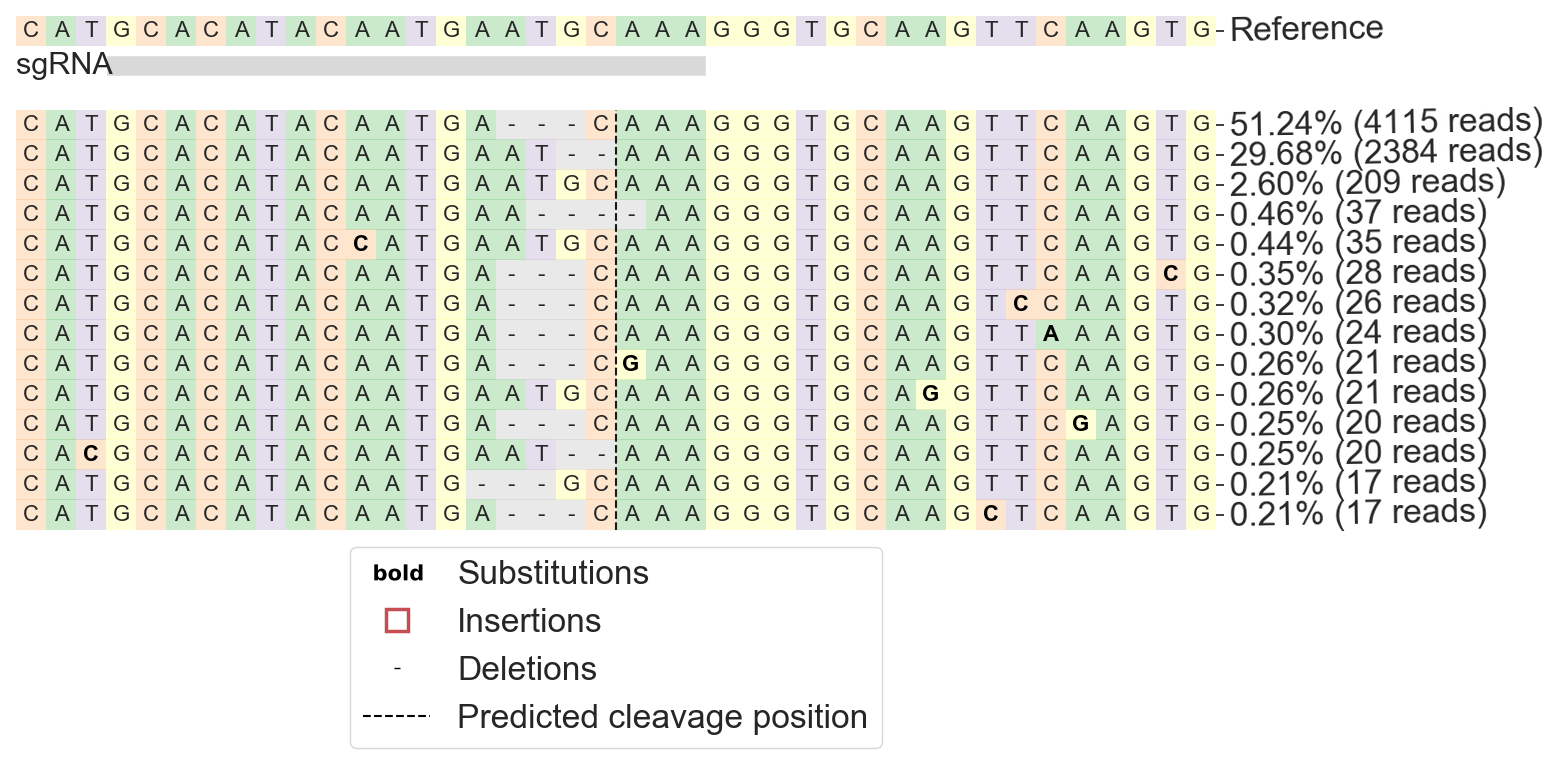 |  |  |  |  |
| S-*epfl9KO14* | 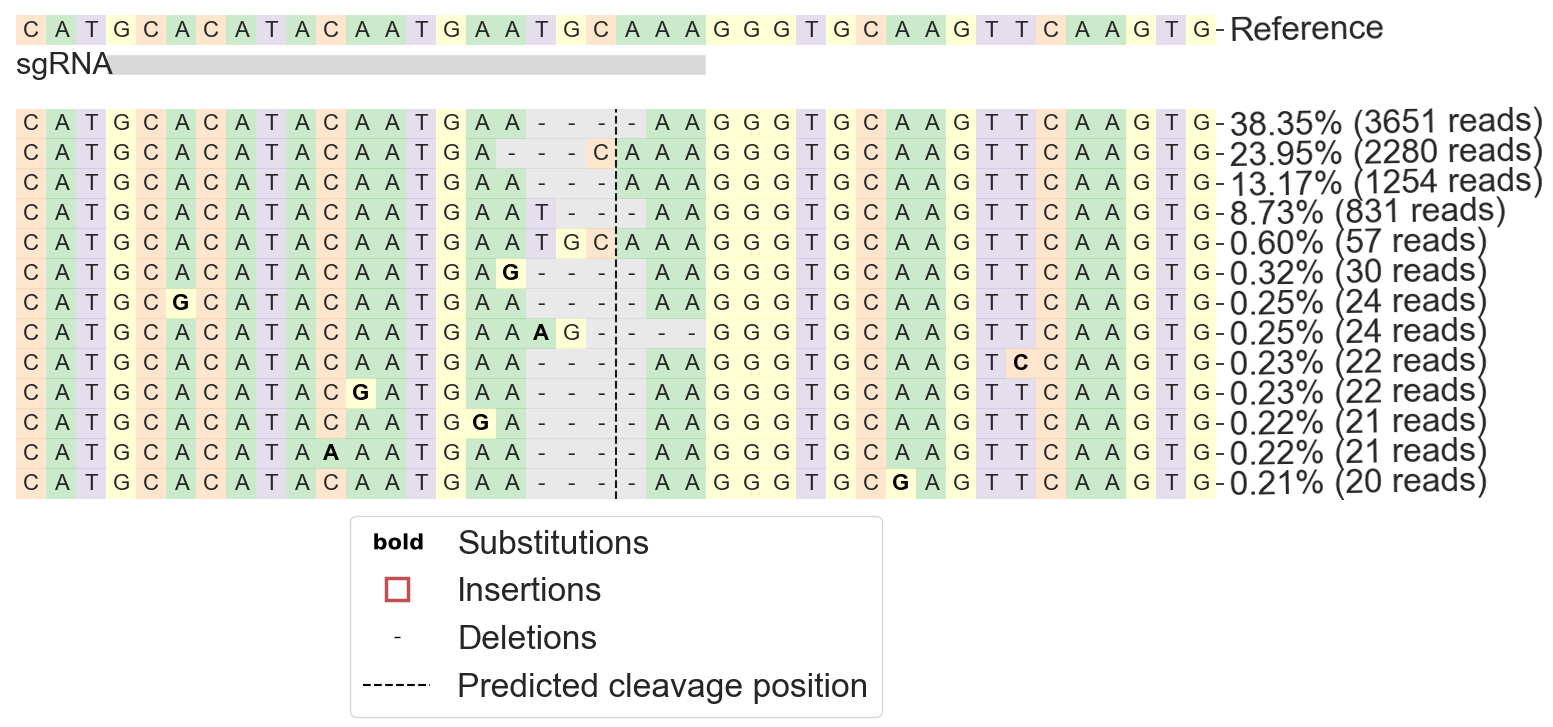 |  |  |  |  |
