# Supplementary material for: VvEPFL9-1 Knock-Out via CRISPR/Cas9 Reduces Stomatal Density in Grapevine
Source: Front Plant Sci. 2022 May 17;13:878001. doi: 10.3389/fpls.2022.878001 (PMC9152544; doi:10.3389/fpls.2022.878001)
Supplement: Supplementary file 5 [file Table_5.DOCX]

**Supplementary Table 5.** One-way ANOVA outputs for gas-exchange analysis collected at different days of water stress application (with reference to **Figure 7**).

| Day 3 : | Df Sum Sq Mean Sq F value Pr(>F) |  |  |  |  |
| --- | --- | --- | --- | --- | --- |
|  | line 2 0.001135 0.0005674 3.727 0.0662 . |  |  |  |  |
|  | Residuals 9 0.001370 0.0001522 |  |  |  |  |
|  |  |  |  |  |  |
| Day 5: | Df Sum Sq Mean Sq F value Pr(>F) |  |  |  |  |
|  | line 2 0.002864 0.0014318 4.792 0.0276 * |  |  |  |  |
|  | Residuals 13 0.003884 0.0002988 |  |  |  |  |
|  |  |  |  |  |  |
|  |  |  |  |  |  |
| Day 7: | Df Sum Sq Mean Sq F value Pr(>F) |  |  |  |  |
|  | line 2 0.0001901 9.506e-05 0.928 0.427 |  |  |  |  |
|  | Residuals 10 0.0010240 1.024e-04 |  |  |  |  |
|  |  |  |  |  |  |
| Day 9: | Df Sum Sq Mean Sq F value Pr(>F) |  |  |  |  |
|  | line 2 0.0000942 4.71e-05 0.304 0.744 |  |  |  |  |
|  | Residuals 11 0.0017051 1.55e-04 |  |  |  |  |
|  |  |  |  |  |  |
| Day 12: | Df Sum Sq Mean Sq F value Pr(>F) |  |  |  |  |
|  | line 2 0.0000485 2.423e-05 0.357 0.709 |  |  |  |  |
|  | Residuals 9 0.0006112 6.791e-05 |  |  |  |  |

**Figure 7A Figure 7B**

| Day 3 : | Df Sum Sq Mean Sq F value Pr(>F) |  |  |  |  |
| --- | --- | --- | --- | --- | --- |
|  | line 2 0.985 0.492 0.15 0.863 |  |  |  |  |
|  | Residuals 9 29.644 3.294 |  |  |  |  |
|  |  |  |  |  |  |
| Day 5: | Df Sum Sq Mean Sq F value Pr(>F) |  |  |  |  |
|  | line 2 9.48 4.739 1.885 0.191 |  |  |  |  |
|  | Residuals 13 32.69 2.515 |  |  |  |  |
|  |  |  |  |  |  |
|  |  |  |  |  |  |
| Day 7: | Df Sum Sq Mean Sq F value Pr(>F) |  |  |  |  |
|  | line 2 3.843 1.9215 2.402 0.141 |  |  |  |  |
|  | Residuals 10 7.998 0.7998 |  |  |  |  |
|  |  |  |  |  |  |
| Day 9: | Df Sum Sq Mean Sq F value Pr(>F) |  |  |  |  |
|  | line 2 3.783 1.891 1.37 0.294 |  |  |  |  |
|  | Residuals 11 15.189 1.381 |  |  |  |  |
|  |  |  |  |  |  |
| Day 12: | Df Sum Sq Mean Sq F value Pr(>F) |  |  |  |  |
|  | line 2 1.127 0.5633 0.767 0.493 |  |  |  |  |
|  | Residuals 9 6.614 0.7349 |  |  |  |  |

**Figure 7C**

| Day 3 : | Df Sum Sq Mean Sq F value Pr(>F) |  |  |  |
| --- | --- | --- | --- | --- |
|  | line 2 16041 8021 3.495 0.0753 . |  |  |  |
|  | Residuals 9 20651 2295 |  |  |  |
|  |  |  |  |  |
| Day 5: | Df Sum Sq Mean Sq F value Pr(>F) |  |  |  |
|  | line 2 6671 3335 3.305 0.0691 . |  |  |  |
|  | Residuals 13 13119 1009 |  |  |  |
|  |  |  |  |  |
| Day 7: | Df Sum Sq Mean Sq F value Pr(>F) |  |  |  |
|  | line 1 4458 4458 1.751 0.234 |  |  |  |
|  | Residuals 6 15277 2546 |  |  |  |
|  |  |  |  |  |
| Day 9: | Df Sum Sq Mean Sq F value Pr(>F) |  |  |  |
|  | line 2 3442 1721.0 3.221 0.0792 . |  |  |  |
|  | Residuals 11 5876 534.2 |  |  |  |
|  |  |  |  |  |
| Day 12: | Df Sum Sq Mean Sq F value Pr(>F) |  |  |  |
|  | line 2 37 18.6 0.014 0.986 |  |  |  |
|  | Residuals 9 11763 1307.0 |  |  |  |
|  |  |  |  |  |
