# Supplementary material for: VvEPFL9-1 Knock-Out via CRISPR/Cas9 Reduces Stomatal Density in Grapevine
Source: Front Plant Sci. 2022 May 17;13:878001. doi: 10.3389/fpls.2022.878001 (PMC9152544; doi:10.3389/fpls.2022.878001)

**Supplementary Figure 1.** Environmental data for the growth chamber and greenhouse during plant phenotyping. **(A)** Temperature and **(B)** humidity were recorded inside the growth chamber from October 29/2020- to November 17/2020 (grey shading) and in greenhouse from November 18- to December 14/2020. Red dotted line indicated the starting of the water stress experiment when the plastic and aluminum coverings were removed from the plants in well-watered conditions to begin the natural dry down.

**B**

**A**


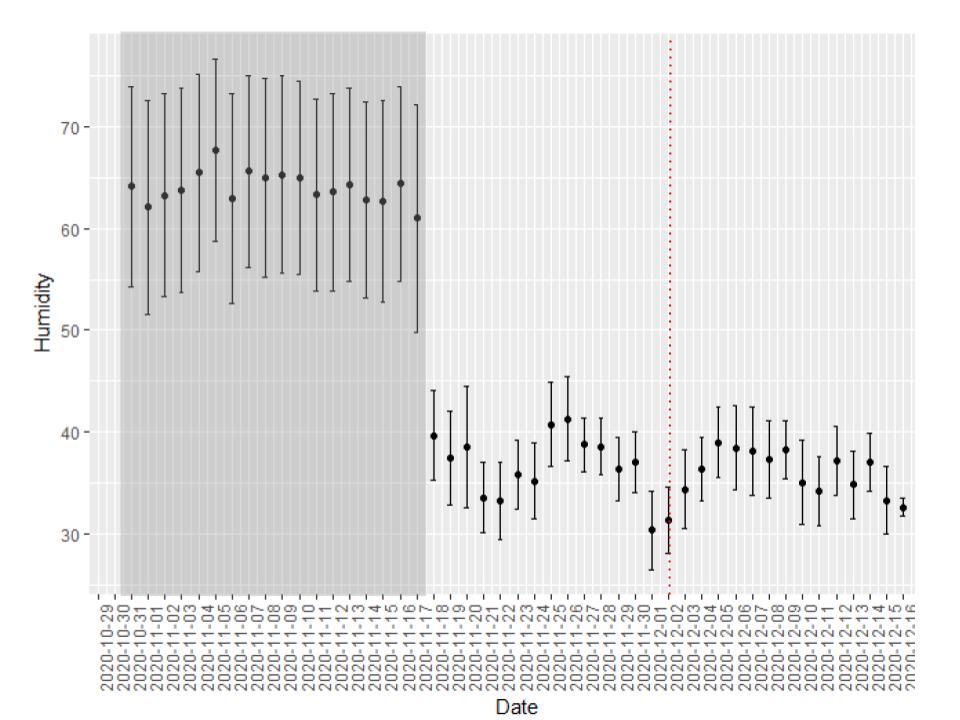

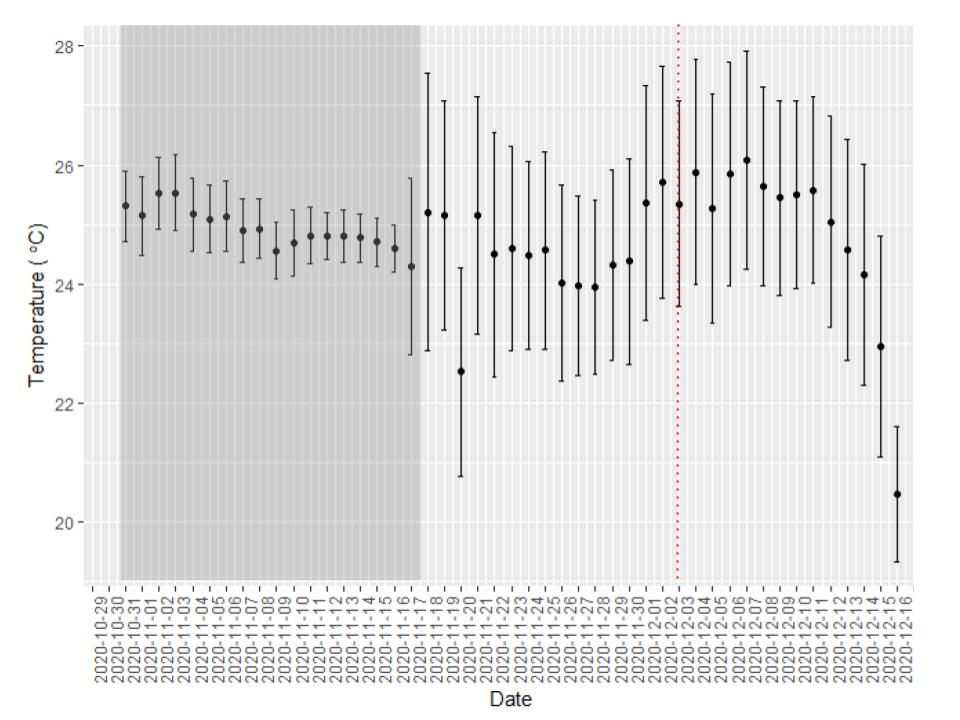

Supplement: Supplementary file 6 [file Data_Sheet_1.DOCX]
