# Supplementary material for: VvEPFL9-1 Knock-Out via CRISPR/Cas9 Reduces Stomatal Density in Grapevine
Source: Front Plant Sci. 2022 May 17;13:878001. doi: 10.3389/fpls.2022.878001 (PMC9152544; doi:10.3389/fpls.2022.878001)

**Supplementary Figure 2.** Method for measuring transpiration in Experiment 1. Aluminum foil and an additional plastic bag were used to prevent any water loss through evaporation. Plants were weighed at the same time daily to measure water lost through transpiration. 133-3 is *S-epfl9KO2*.


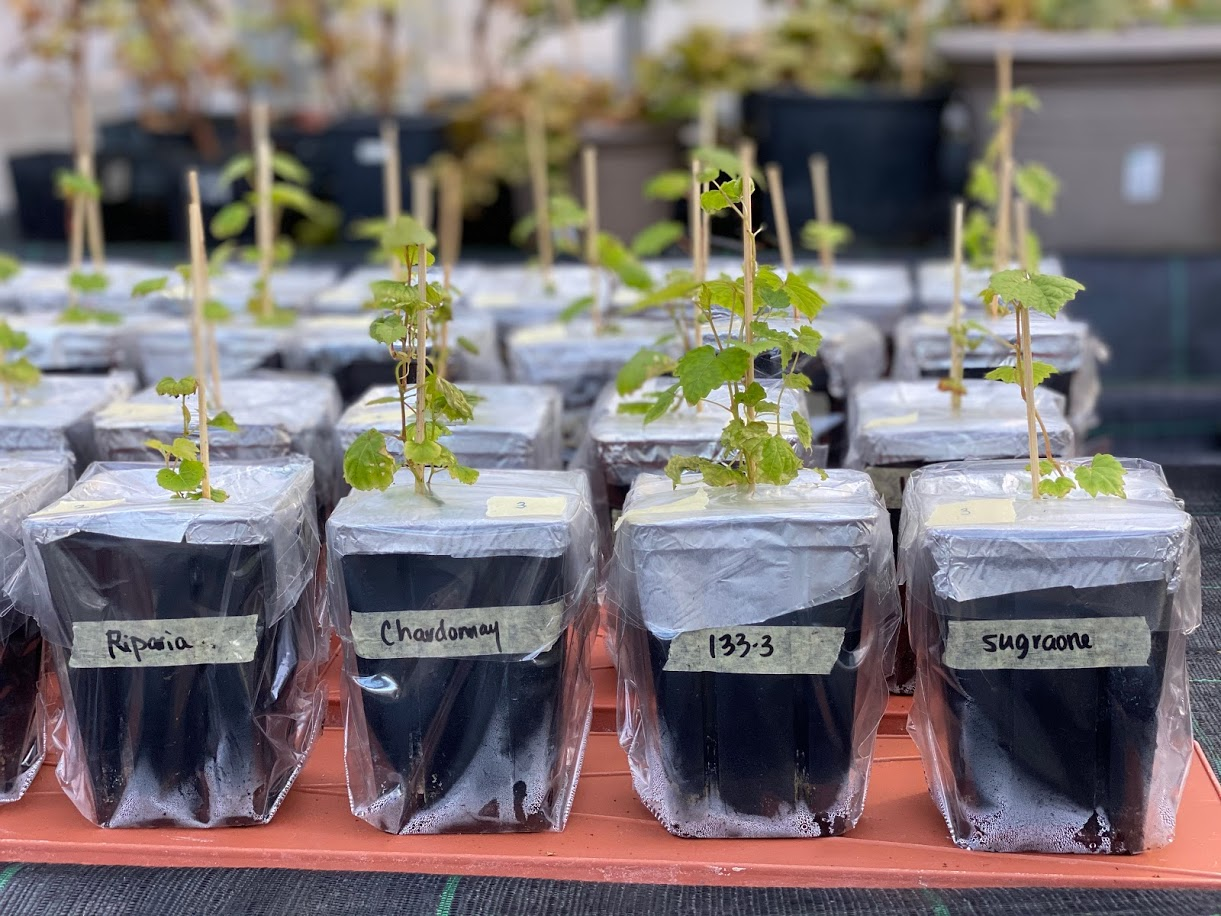

Supplement: Supplementary file 7 [file Data_Sheet_2.DOCX]
