# Supplementary material for: VvEPFL9-1 Knock-Out via CRISPR/Cas9 Reduces Stomatal Density in Grapevine
Source: Front Plant Sci. 2022 May 17;13:878001. doi: 10.3389/fpls.2022.878001 (PMC9152544; doi:10.3389/fpls.2022.878001)

**Supplementary Figure 3.** Fraction of Transpirable Soil Water (FTSW) for days 1-11 of well-watered conditions and days 12-27 of the natural dry-down. The red vertical line indicated the timing of unwrapping the pots to begin the natural dry down, after full re-watering based on initial pot weights. The average FTSW is shown by the point, with the standard error indicated by the whiskers.


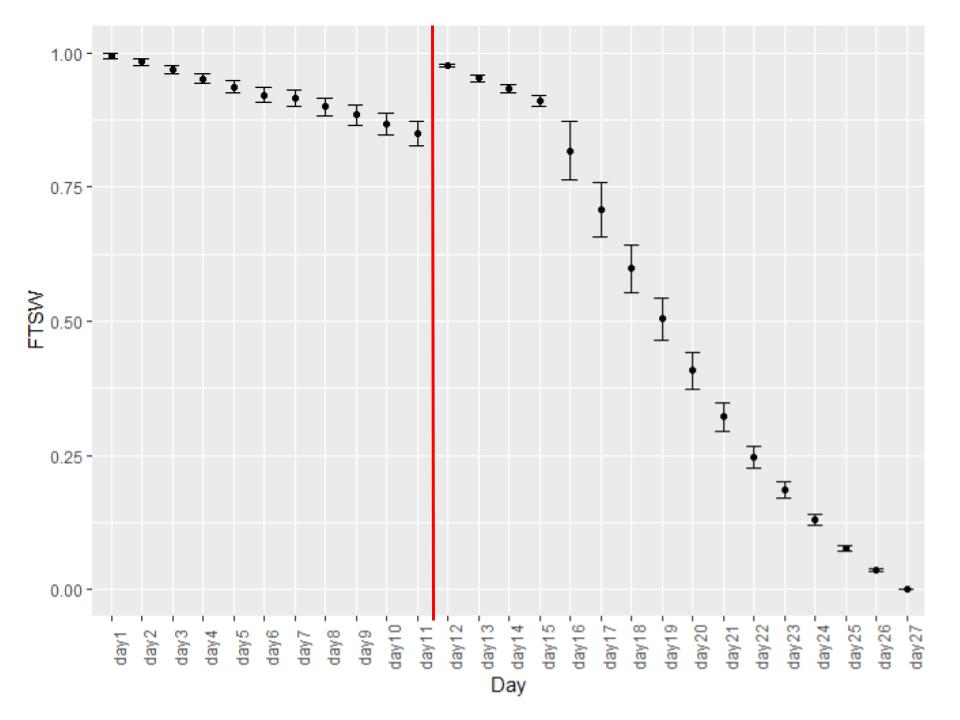

Supplement: Supplementary file 8 [file Data_Sheet_3.DOCX]
