# Supplementary material for: VvEPFL9-1 Knock-Out via CRISPR/Cas9 Reduces Stomatal Density in Grapevine
Source: Front Plant Sci. 2022 May 17;13:878001. doi: 10.3389/fpls.2022.878001 (PMC9152544; doi:10.3389/fpls.2022.878001)
Supplement: Supplementary file 9 [file Data_Sheet_4.DOCX]

**Supplementary Figure 4.** Method for assessing leaf area. (**A**) In Experiments 1 and 2, plants were photographed at the same distance and angle, and compared to the same standard area (square in red=4 cm^2^) using Easy Leaf Area software to retrieve total relative leaf area. 133-3 is *S-epfl9KO2*. (**B**) In Experiment 3, for each biological replicate, a projected leaf area (pixels) was calculated as the average green pixels in four RGB imaging automatically collected at different pot angles and analyzed with the WIWAM software in the automated phenotyping platform (WIWAM, Gand, Belgium) at the Plant Phenotyping Facility of Fondazione Edmund Mach.


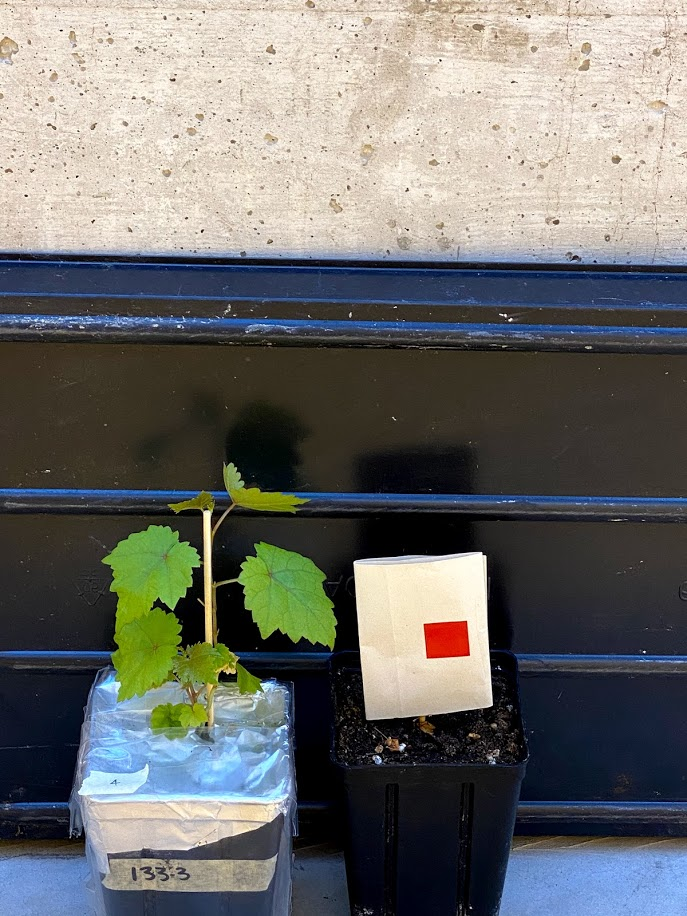


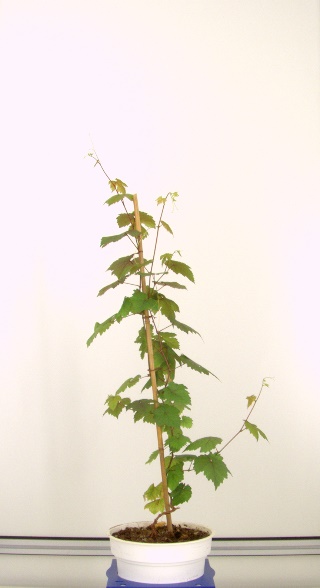

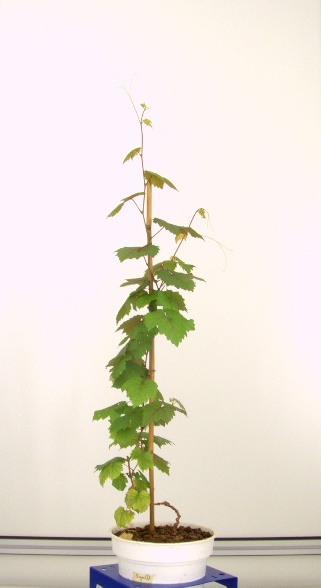

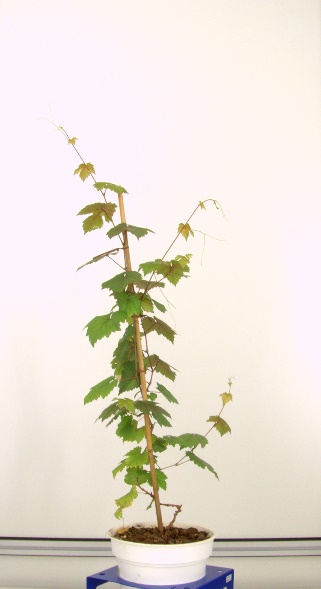

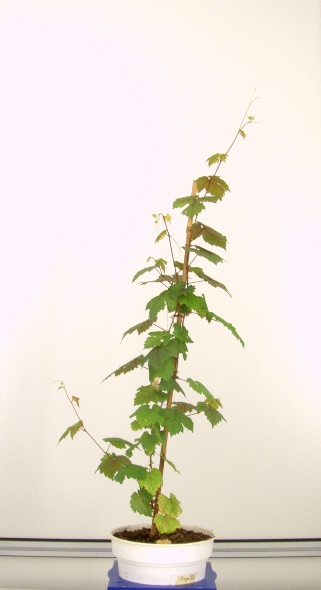


**B**

**A**
