# Supplementary material for: VvEPFL9-1 Knock-Out via CRISPR/Cas9 Reduces Stomatal Density in Grapevine
Source: Front Plant Sci. 2022 May 17;13:878001. doi: 10.3389/fpls.2022.878001 (PMC9152544; doi:10.3389/fpls.2022.878001)
Supplement: Supplementary file 10 [file Data_Sheet_5.DOCX]

**Supplementary Figure 5.** Estimation of the relationship between non-destructive and destructive biomass estimation methods. **(A)** Leaf area estimate via RGB imaging (45° angle) *versus* leaf area estimated via RGB imaging (90° angle). **(B)** Leaf area estimate via RGB imaging (45° angle) *versus* fresh weight. **(C)** Leaf area estimate via RGB imaging (45° angle) *versus* and dry weight. **(D)** Fresh weight *versus* dry weight. Leaf area was calculated using Easy Leaf Area (Ealson and Bloom 2014). Linear regression was significant (p<0.05) for all the correlations (R^2^ between 0.82 and 0.89) thus validating the non-destructive approach used in this work to estimate leaf area accumulation dynamics.


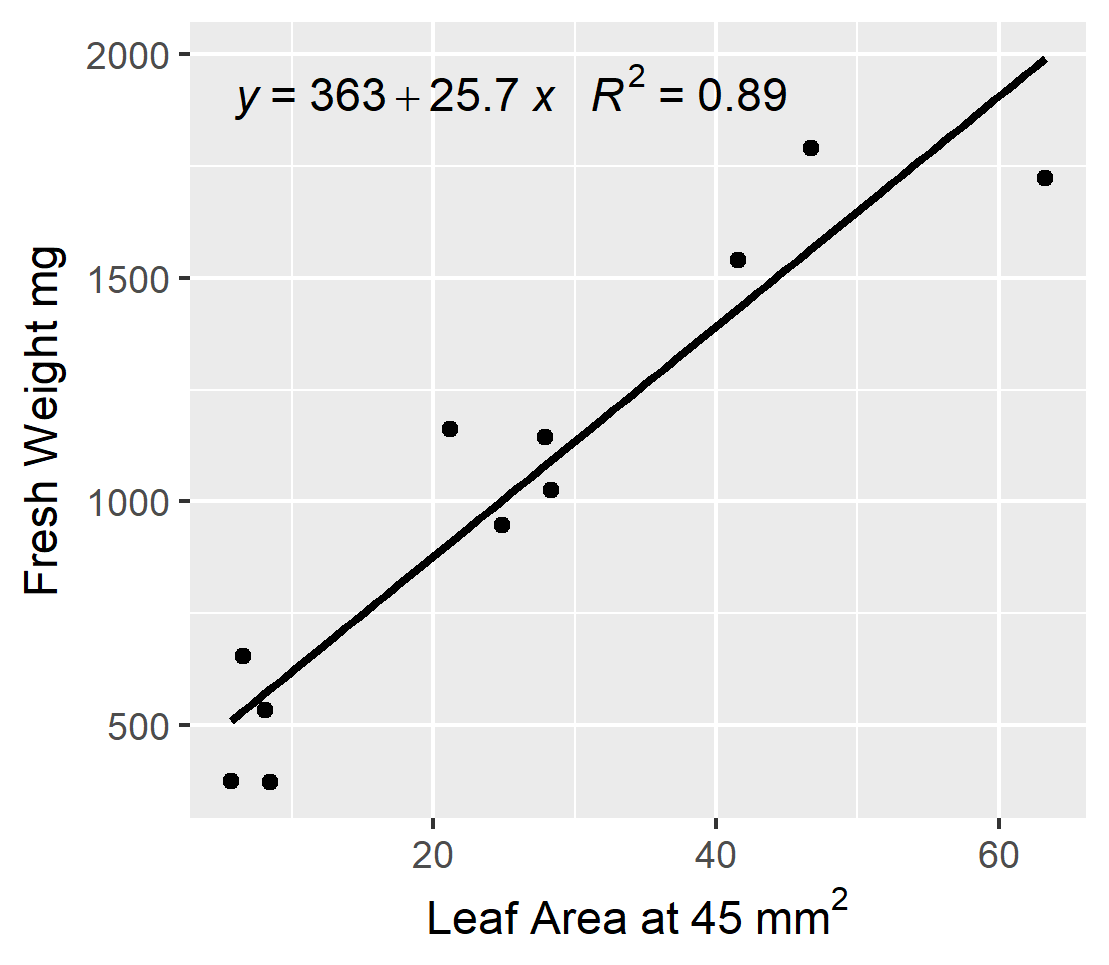

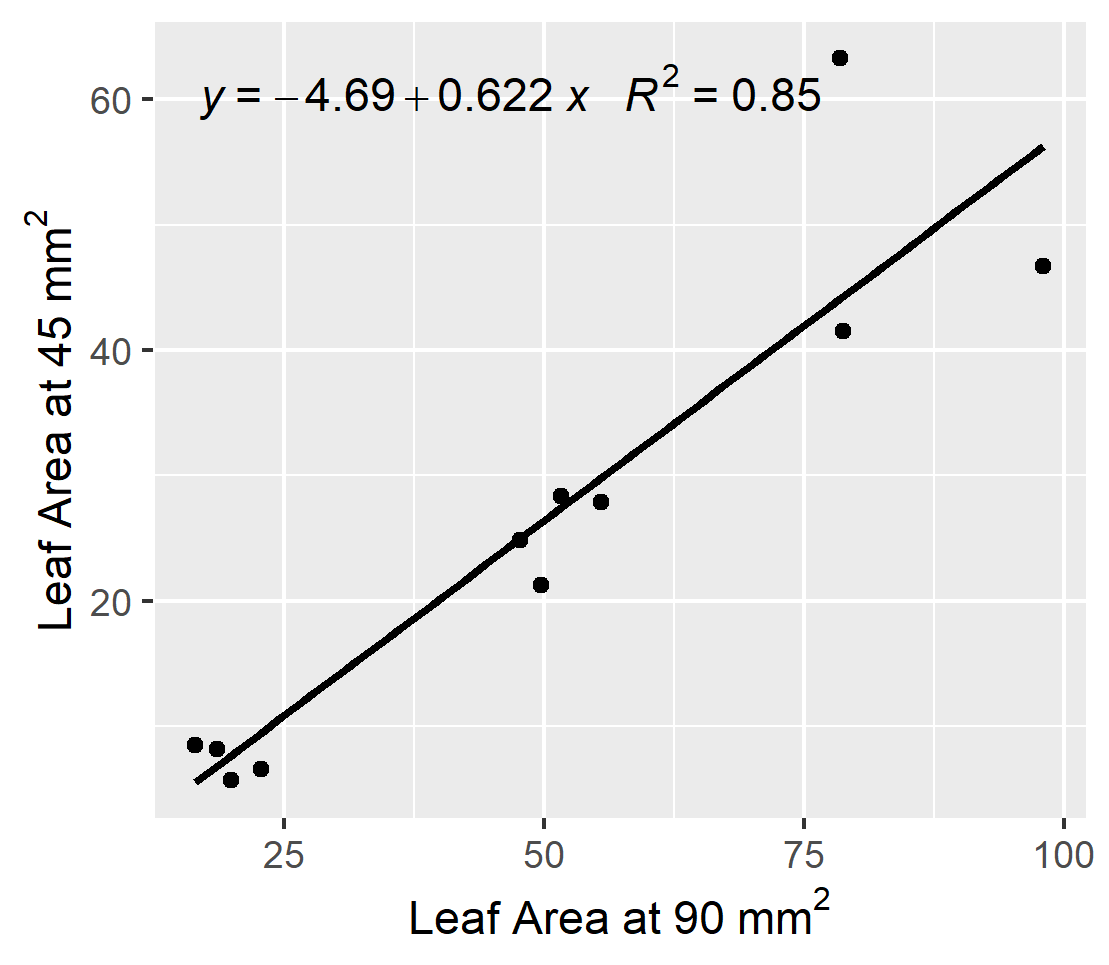


**B**

**A**


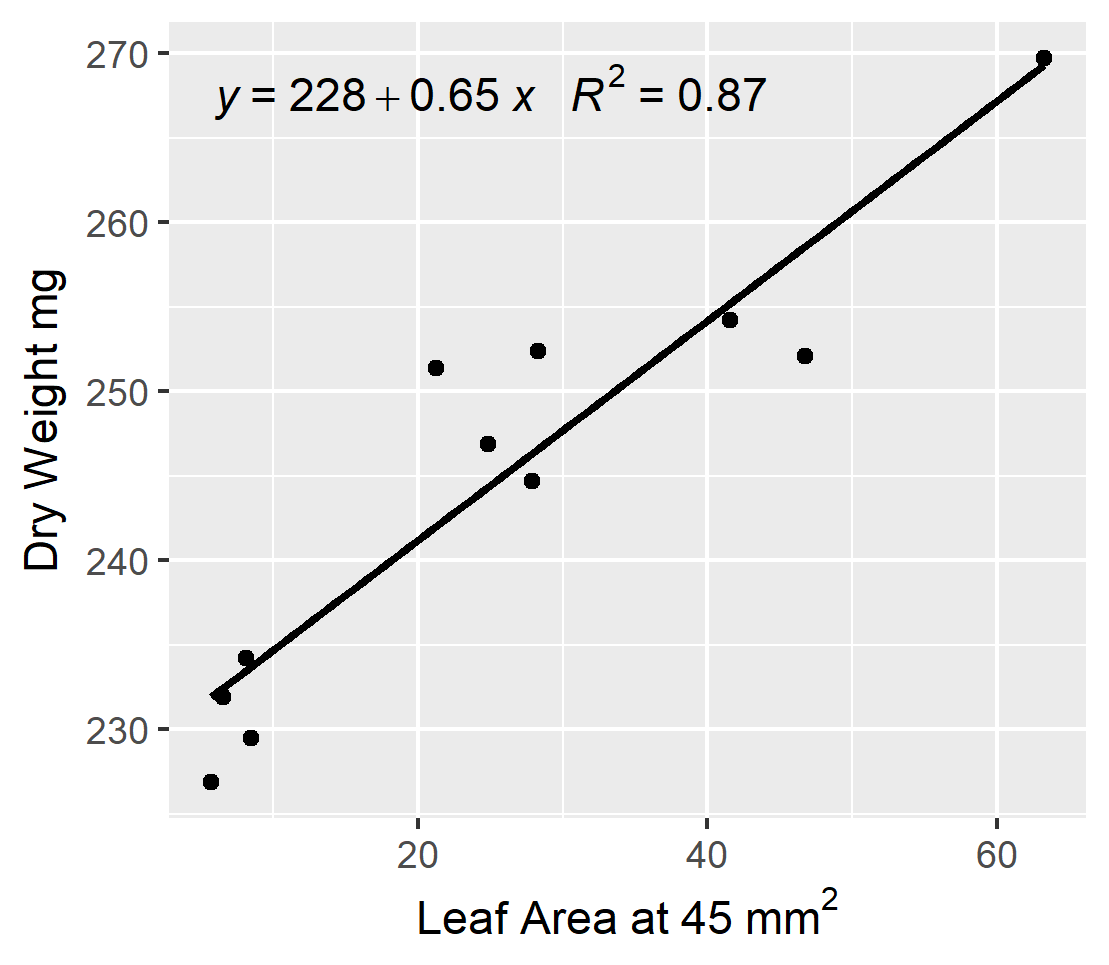

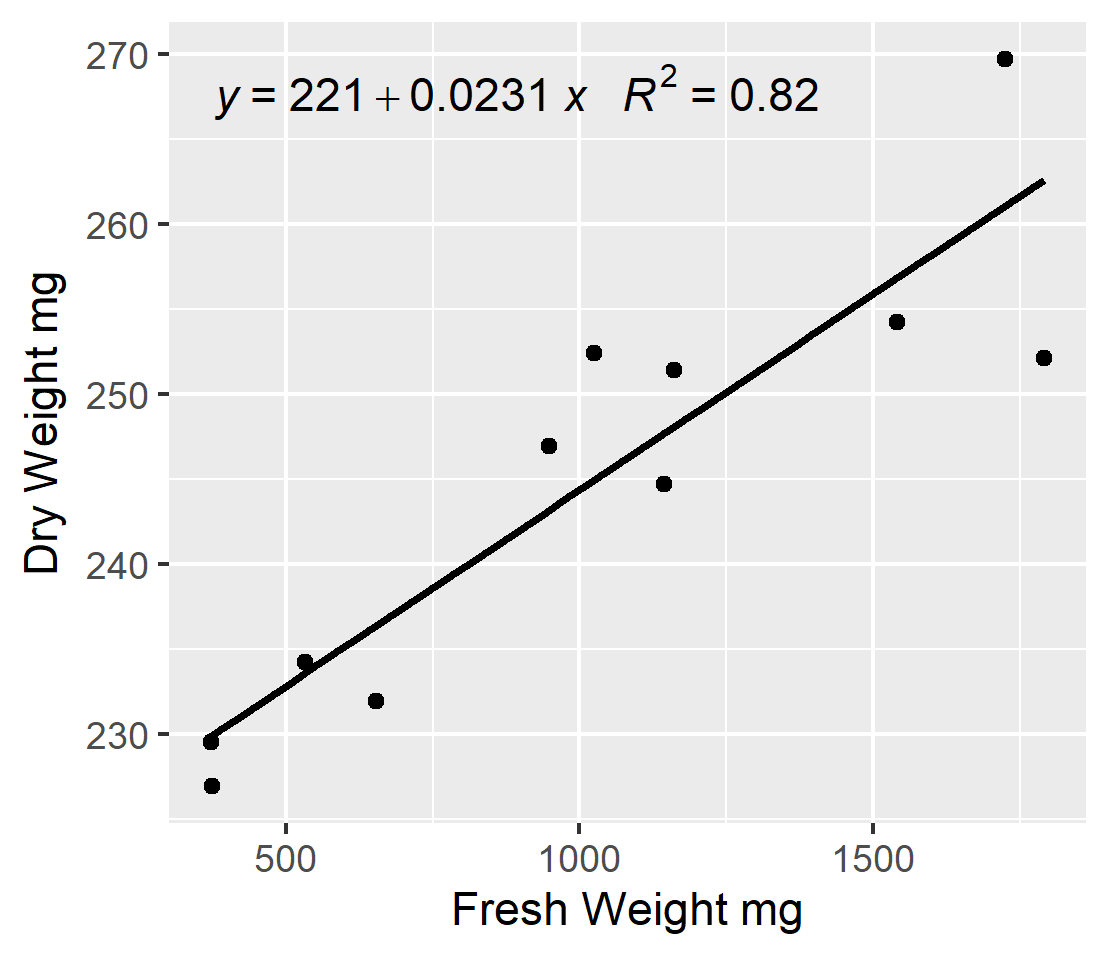


**C**

**D**
