# Supplementary material for: VvEPFL9-1 Knock-Out via CRISPR/Cas9 Reduces Stomatal Density in Grapevine
Source: Front Plant Sci. 2022 May 17;13:878001. doi: 10.3389/fpls.2022.878001 (PMC9152544; doi:10.3389/fpls.2022.878001)
Supplement: Supplementary file 11 [file Data_Sheet_6.DOCX]

**
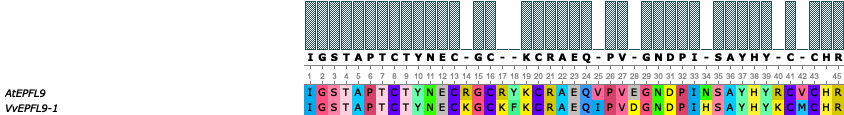

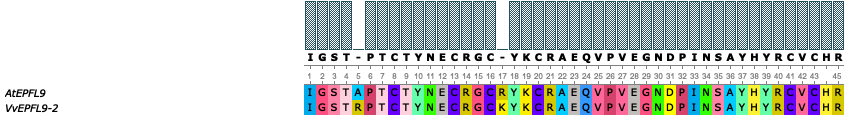
Supplementary Figure 6.** Alignment of *AtEPFL9* against *VvEPF9-1* and *VvEPF9-2*. The alignment was made using Unipro UGENE software (Okonechnikov et al., 2012). The red rectangle indicates the designed target site for the CRISPR/Cas9 system.

***AtEPFL9***

***VvEPFL9-1***

***AtEPFL9***

***VvEPFL9-2***

Okonechnikov, K. *et al.* Unipro UGENE: a unified bioinformatics toolkit. *Bioinformatics* **28**, 1166–1167 (2012).
