# Supplementary material for: VvEPFL9-1 Knock-Out via CRISPR/Cas9 Reduces Stomatal Density in Grapevine
Source: Front Plant Sci. 2022 May 17;13:878001. doi: 10.3389/fpls.2022.878001 (PMC9152544; doi:10.3389/fpls.2022.878001)
Supplement: Supplementary file 12 [file Data_Sheet_7.DOCX]

**Supplementary Figure 7.** Analysis of CRISPR/Cas9 editing in the potential “off-target” site in *VvEPFL9-2* in nine transgenic lines

| **Lines** | **Sanger sequencing** |
| --- | --- |
|  | Reference sequence (wt) of the off-target site in *VvEPFL9-2*  GCACTTACAATGAATGTAGA |
| S-*epfl9KO1* | 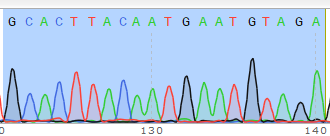 |
| S-*epfl9KO2* | 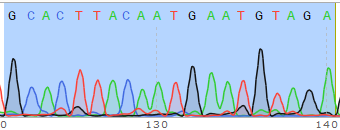 |
| S-*epfl9KO3* | 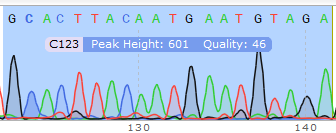 |
| S-*epfl9KO4* | 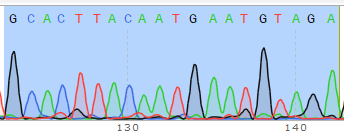 |
| S-*epfl9KO5* | 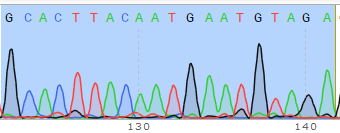 |
| S-*epfl9KO6* | 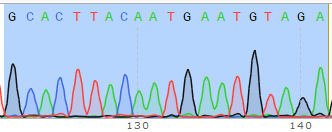 |
| S-*epfl9KO7* | 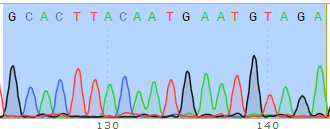 |
| S-*epfl9KO8* | 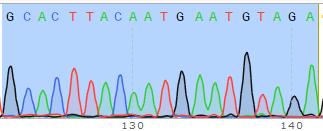 |
| S-*epfl9KO9* | 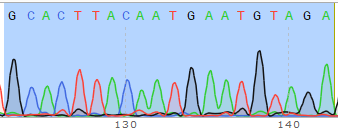 |
|  |  |
