# Supplementary material for: VvEPFL9-1 Knock-Out via CRISPR/Cas9 Reduces Stomatal Density in Grapevine
Source: Front Plant Sci. 2022 May 17;13:878001. doi: 10.3389/fpls.2022.878001 (PMC9152544; doi:10.3389/fpls.2022.878001)

**Supplementary Figure 8.** Example of photosynthetic CO_2_ response curves (*A*/*Ci*). Curves were carried out in Sugraone (**A**), *S-epfl9KO1* (**B**) and *S-epfl9KO2* (**C**). Fitted limiting rate for photosynthesis is shown as black line. Colored lines indicate the two photosynthesis rates of the model (Ac and Aj). **D** an example of light curve for *S-epfl9KO2* is shown. PPFD= Photosynthetic Photon Flux Density.


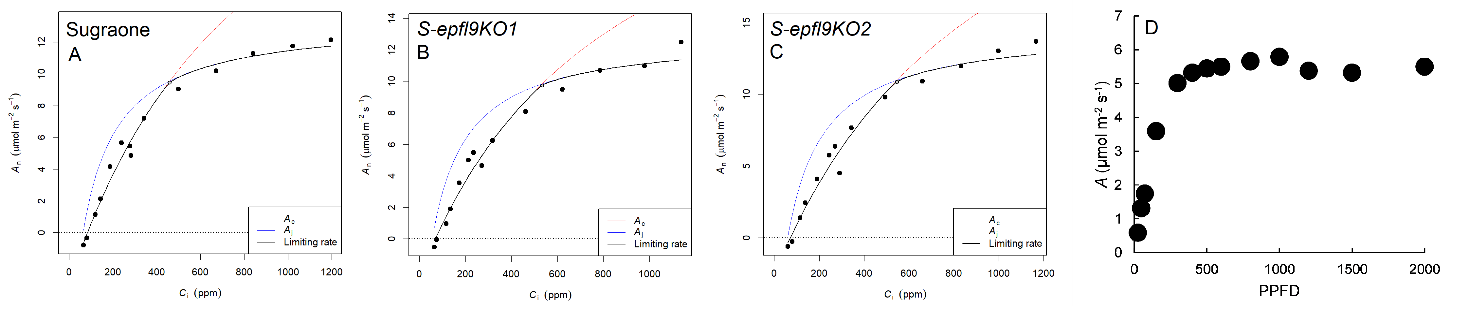

Supplement: Supplementary file 13 [file Data_Sheet_8.DOCX]
