# Supplementary material for: VvEPFL9-1 Knock-Out via CRISPR/Cas9 Reduces Stomatal Density in Grapevine
Source: Front Plant Sci. 2022 May 17;13:878001. doi: 10.3389/fpls.2022.878001 (PMC9152544; doi:10.3389/fpls.2022.878001)

**Supplementary Figure 9.** Carbon Isotope discrimination (δ13C) of grapevine leaves assessed after the water stress treatment. Whiskers indicate the ranges of the minimum and maximum values and data were analyzed with one-way ANOVA (n=3-6). Different letters indicate significantly different values according to Fisher’s test. Data were collected on fully expanded leaves.


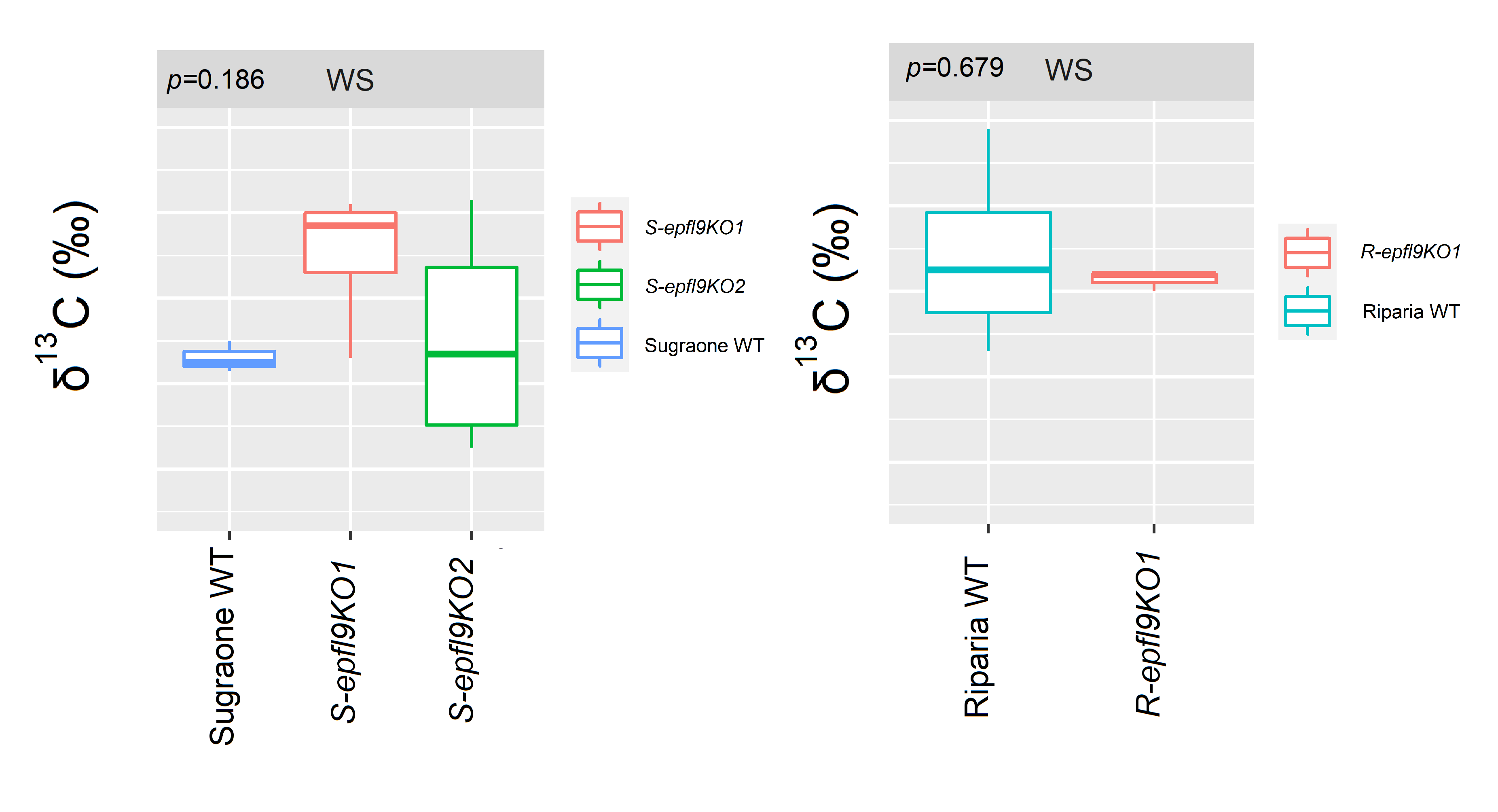

Supplement: Supplementary file 14 [file Data_Sheet_9.DOCX]
